# Citicoline and Coenzyme Q10: Therapeutic Agents for Glial Activation Reduction in Ocular Hypertension

**DOI:** 10.3390/ph18050694

**Published:** 2025-05-08

**Authors:** José A. Matamoros, Sara Rubio-Casado, José A. Fernández-Albarral, Miguel A. Martínez-López, Ana I. Ramírez, Elena Salobrar-García, Eva M. Marco, Victor Paleo-García, Rosa de Hoz, Inés López-Cuenca, Lorena Elvira-Hurtado, Lidia Sánchez-Puebla, José M. Ramírez, Meritxell López-Gallardo, Juan J. Salazar

**Affiliations:** 1 Ramon Castroviejo Institute for Ophthalmic Research, Complutense University of Madrid (ROR 02p0gd045), 28040 Madrid, Spain; jomatamo@ucm.es (J.A.M.); srubio02@ucm.es (S.R.-C.); joseaf08@ucm.es (J.A.F.-A.); miguma19@ucm.es (M.A.M.-L.); airamirez@med.ucm.es (A.I.R.); elenasalobrar@med.ucm.es (E.S.-G.); emmarco@ucm.es (E.M.M.); rdehoz@med.ucm.es (R.d.H.); inelopez@ucm.es (I.L.-C.); marelvir@ucm.es (L.E.-H.); lidsan02@ucm.es (L.S.-P.); ramirezs@med.ucm.es (J.M.R.); 2 Health Research Institute of the Hospital Clínico San Carlos (IdISSC) (ROR 014v12a39), 28040 Madrid, Spain; 3 Department of Immunology, Ophthalmology and ENT, Faculty of Optics and Optometry, Complutense University of Madrid, 28040 Madrid, Spain; 4 Department of Immunology, Ophthalmology and ENT, School of Medicine, Complutense University of Madrid, 28040 Madrid, Spain; 5 Department of Genetics, Microbiology and Physiology, Faculty of Biological Sciences, Complutense University of Madrid, 28040 Madrid, Spain; 6 Department of Physiology, School of Medicine, Complutense University of Madrid, 28040 Madrid, Spain; vipaleo@ucm.es

**Keywords:** ocular hypertension, retina, visual pathway, glial cells, Citicoline, Coenzyme Q10, glaucoma, microglia, astrocytes

## Abstract

**Background/Objectives**: The loss of retinal ganglion cells (RGCs) is a hallmark of glaucoma, a major cause of blindness. Glial cell activation due to increased intraocular pressure (IOP) significantly contributes to RGC death. Therefore, substances with anti-inflammatory properties could help prevent that process. This study investigated whether combining Citicoline and Coenzyme Q10 (CoQ10) can reduce glial activation in the retina and the rest of the visual pathway, potentially preventing neurodegeneration in a mouse model of unilateral laser-induced ocular hypertension (OHT). **Methods:** Four groups of mice were used: vehicle (n = 12), CitiQ10 (n = 12), OHT–vehicle (n = 18), and OHT–CitiQ10 (n = 18). The administration of Citicoline and CoQ10 was performed orally once a day, initiated 15 days prior to the laser treatment and maintained post-treatment until sacrifice (3 days for retina or 7 days for the rest of the visual pathway). The retina, dorsolateral geniculate nucleus, superior colliculus, and visual cortex (V1) were immunohistochemically stained and analyzed. **Results:** In the laser–CitiQ10 group, the Citicoline + CoQ10 compound revealed (1) an IOP decrease at 24 h and 3 days post-laser; and (2) reduced signs of macroglial (decreased GFAP area) and microglial (soma size, arbor area, microglia number, P2RY12 expression) activation in the retina and in the rest of the visual pathway (reduced activated microglial phenotypes and lower GFAP expression). **Conclusions:** This study shows that oral administration of Citicoline and CoQ10 can reduce glial activation caused by increased IOP in retina and visual pathway in a mouse model of OHT, potentially protecting RGCs from OHT-induced inflammation.

## 1. Introduction

One of the primary causes of irreversible blindness around the world is glaucoma [1]. It is a neurodegenerative disease characterized by the death of retinal ganglion cells (RGCs), leading to progressive visual field loss [2]. This neurodegenerative process not only affects the retina and optic nerve, where the RGCs and their axons are located, but also extends to higher visual centers [3,4,5,6,7]. In glaucoma patients, a decrease in the grey matter in the primary visual cortex and a reduction in the volume occupied by the lateral geniculate nucleus (LGN) have been observed [8]. In rodents, the main centers affected by ocular hypertension (OHT) are the LGN and the superior colliculus (SC), where 90% of the RGCs project in mice [3].

The primary risk factor for glaucoma is elevated intraocular pressure (IOP), and most current treatments aim to control it [9]. However, sometimes, despite the normalization of IOP, the neurodegenerative process continues, suggesting the existence of other factors influencing RGC death [9]. Neuroinflammation is one of those factors, indicating the involvement of the immune system in glaucoma and its progression, as in other neurodegenerative diseases [10,11]. Immunoregulation in the retina is carried out by glial cells (astrocytes, Müller glia, and microglia) [12,13], and increased IOP can cause the activation of retinal and optic nerve glial cells. If this activation becomes chronic, it can lead to a pro-inflammatory state in the retina, potentially disrupting the blood–retinal barrier and inducing RGC death [14], and this pro-inflammatory state could extend to the rest of the visual pathway. Given that glaucoma is a neurodegenerative pathology and that it has also been shown to affect the contralateral eye to the one initially damaged [15,16], it is quite possible that the damage could be transmitted by neuroinflammatory processes across the entire visual system. On the other hand, the loss of RGCs, whose axons constitute the optic nerve, could involve the loss of synaptic connections and, as a consequence, the neurons’ degeneration in the different relay nuclei of the visual pathway [17], such as SC, the dorsolateral geniculate nucleus (dLGN), and the primary visual cortex (V1).

Glial activation has been demonstrated in humans and in both hereditary and induced glaucoma models [15,18,19,20]. In these models, astrocyte and Müller glia activation leads to significant biochemical, functional, and morphological changes, including cell body thickening, an increased number and length of cellular processes, and up-regulation of cytoskeletal proteins such as GFAP, nestin, and vimentin, which are key markers of this activation process [21,22]. Additionally, microglial activation involves an increase in their number, migration to the site of damage, morphological changes such as soma thickening, retraction and reorientation of processes, and the adoption of amoeboid forms with high phagocytic capacity [23]. Furthermore, activated microglia have the ability to change the expression of different receptors (CD200R, CX3CR1, and P2RY12) and genes, promoting the synthesis and release of inflammatory cytokines (IL-6, TNF-α, and IL-1β, among others) [12,23,24,25]. These cytokines could contribute to RGCs’ death via both intrinsic and extrinsic pathways, thereby worsening the neurodegenerative process [26,27]. Therefore, controlling the inflammatory process could reduce the neurodegenerative process in glaucomatous pathology.

Currently, various compounds are being tested to reduce the neurodegenerative process associated with glaucoma, including Citicoline and Coenzyme Q10 (CoQ10). In a previous study, we demonstrated the neuroprotective effect of the combination of these two compounds in a unilateral glaucoma animal model induced by a laser [7]. However, there are no studies on the anti-inflammatory capacity of the combination of these substances in relation to glaucoma. Moreover, there are very few studies that analyze the anti-inflammatory effects of Citicoline or CoQ10 separately in animal models of glaucoma. Regarding Coenzyme Q10, only four studies exist, each using different experimental models of glaucoma: a genetic model with DBA/2j mice [28], an ischemia–reperfusion model in mice [29] or rats [30], and a mechanical optic nerve injury model in rats [31]. All these studies demonstrate an anti-inflammatory effect of Coenzyme Q10, evidenced by the decreased activation of astroglial and microglial cells (due to lower expression of GFAP and Iba-1) [28,29,30,31] and reduced levels of pro-inflammatory cytokines (IL-1β, IL-6, and TNFα) [30]. Regarding Citicoline, there is only one experimental study in a glaucoma model in rats treated with kainate to induce RGC death through excitotoxicity, which examines the anti-inflammatory effect of this compound, finding a reduction in the expression of PERK (a pathway that regulates the production of inflammatory cytokines) in Müller glia and astrocytes. Given the scarcity of studies on the anti-inflammatory effects of these compounds in glaucoma models, in this study, we aimed to investigate whether the combination of CoQ10 and Citicoline could reduce different morphological signs of microglial and macroglial activation in the retina and along the visual pathway, thereby helping to prevent neurodegenerative processes in this unilateral ocular hypertension model induced by a laser.

## 2. Results

### 2.1. Intraocular Pressure: Citicoline + CoQ10 Exerts a Mild Hypotensive Effect in Early Stages Following Ocular Hypertension Induction

In the present study, we analyzed IOP differences across multiple groups of eyes: vehicle; CitiQ10; OHT; contralateral; OHT-CitiQ10; and contralateral-CitiQ10. IOP was measured at various time points: 24 h, 48 h, 3 days, 5 days, and 7 days.

**Figure 1:**
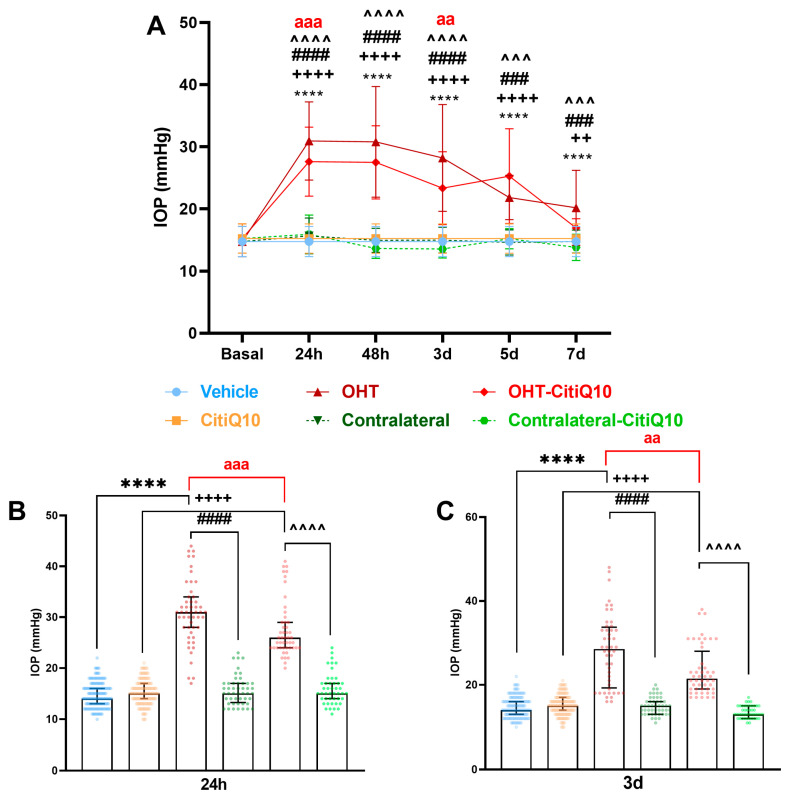
Intraocular pressure (IOP) graphics. (**A**) IOP in different study groups after induction of hypertension. (**B**) IOP data in the different study groups 24 h after OHT induction. (**C**) IOP data in the different study groups 3 d after OHT induction. Data are (**A**) mean ± standard deviation (SD) or (**B**,**C**) median with interquartile range; each data point in the column graph represents a single measurement of IOP. Abbreviations: ocular hypertension (OHT); Citicoline + Coenzyme Q10 (CitiQ10). Statistical significance indicators: **** *p* < 0.0001 vehicle vs. OHT; ++ *p* < 0.01, ++++ *p* < 0.0001, CitiQ10 vs. OHT-CitiQ10; ### *p* < 0.001, #### *p* < 0.0001 OHT vs. contralateral; ^^^ *p* < 0.001, ^^^^ *p* < 0.0001 OHT–CitiQ10 vs. contralateral CitiQ10; aa *p* < 0.01, aaa *p* < 0.001 for OHT vs. OHT-CitiQ10.

At all time points, OHT and OHT-CitiQ10 eyes showed a significant increase in IOP compared to their respective controls (vehicle and CitiQ10) and their contralateral eyes (*p* < 0.0001 in most cases, except *p* < 0.001 for OHT vs. contralateral at 5 days; OHT-CitiQ10 vs. contralateral CitiQ10 at 5 days; OHT vs. contralateral at 7 days; OHT-CitiQ10 vs. contralateral CitiQ10 at 7 days, and *p* < 0.01 for OHT-CitiQ10 vs. CitiQ10 at 7 days) (Figure 1A). These findings suggest that in OHT and OHT-CitiQ10, IOP remains elevated, decreasing by day 7 after OHT induction.

However, when comparing OHT eyes with OHT-CitiQ10 eyes, the latter showed significantly lower IOP values at 24 h (*p* < 0.001) (Figure 1B) and at 3 days (*p* < 0.01) (Figure 1C), indicating that the Citicoline + CoQ10 combination has a slight hypotensive effect in the initial days following OHT induction.

### 2.2. Retinal Glial Cells: The Combination of Citicoline and CoQ10 Reduces the Signs of Glial Activation Produced by Laser-Induced OHT

#### 2.2.1. Morphological Signs of Microglial Activation

##### Microglial Cell Number

In the **outer segment (OS)**, both treated with Citicoline + CoQ10 and untreated OHT eyes showed a significant increase in the number of microglial cells compared to their respective controls (vehicle and CitiQ10) and contralateral counterparts (*p* < 0.0001, in all cases). However, when comparing treated and untreated OHT and contralateral eyes, those treated with Citicoline + CoQ10 showed a significant reduction in microglial cell number in both OHT-CitiQ10 (*p* < 0.0001) and contralateral-CitiQ10 eyes (*p* < 0.05).

**Figure 2:**
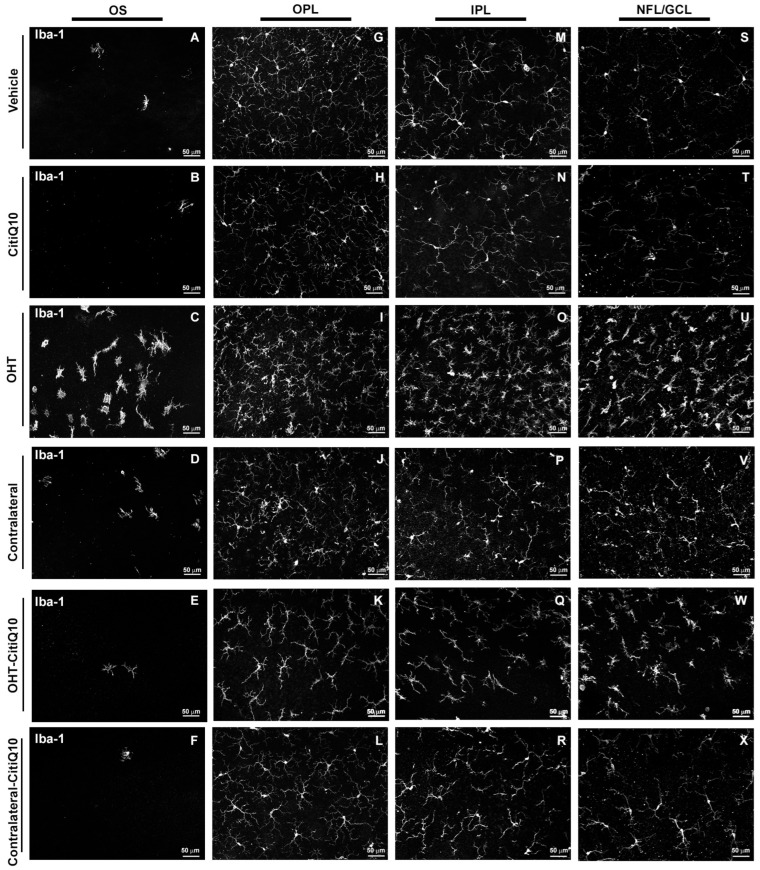
Comparison of Iba-1+ cells in untreated and Citicoline + CoQ10-treated eyes across the different retinal layers: photoreceptor outer segment layer (OS), outer plexiform layer (OPL), inner plexiform layer (IPL), and nerve fiber layer–ganglion cell layer (NFL-GCL), 3 days after ocular hypertension (OHT) induction. The images show retinal whole-mounts with Iba-1 immunostaining.

**Figure 3:**
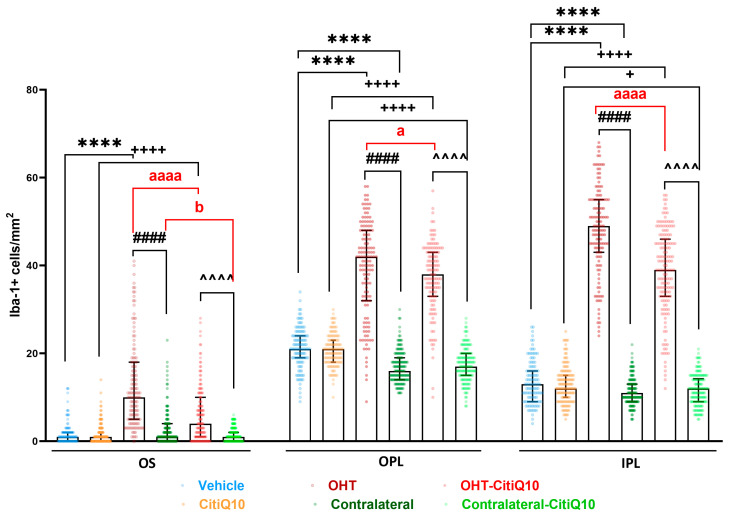
Number of Iba-1+ cells in untreated and Citicoline + CoQ10-treated eyes, 3 days after ocular hypertension (OHT) induction in the photoreceptor outer segment layer (OS), outer plexiform layer (OPL), and inner plexiform layer (IPL). Data are median with interquartile range; each data point in the column graph represents a single measurement of the Iba-1+ cells per area of 0.1502 mm^2^. Statistical significance indicators: **** *p* < 0.0001; vs. Vehicle. + *p* < 0.05, ++++ *p* < 0.0001; vs. CitiQ10. #### *p* < 0.0001; OHT vs. contralateral. ^^^^ *p* < 0.0001; OHT-CitiQ10 vs. contralateral-CitiQ10. a *p* < 0.05, aaaa *p* < 0.0001; OHT vs. OHT-CitiQ10. b *p* < 0.05; contralateral vs. contralateral-CitiQ10.

In the **outer plexiform layer (OPL) and inner plexiform layer (IPL)**, both eyes treated with Citicoline + CoQ10 and untreated OHT eyes exhibited a significant increase in the number of microglial cells compared to their respective controls (vehicle and CitiQ10) and contralateral counterparts (*p* < 0.0001, in all cases). Additionally, both treated and untreated contralateral eyes showed a significant decrease in the number of microglial cells compared to their respective controls (in OPL *p* < 0.0001 both; in IPL contralateral vs. vehicle *p* < 0.0001 and contralateral-CitiQ10 vs. CitiQ10 *p* < 0.05). When comparing treated and untreated OHT eyes, those treated with Citicoline + CoQ10 demonstrated a significant reduction in microglial cell number (in OPL *p* < 0.05; in IPL *p* < 0.0001) (Figure 2 and Figure 3).

##### Area of the Retina Occupied by Iba-1 + Cells (Iba1-RA) in the NFL-GCL

**Figure 4:**
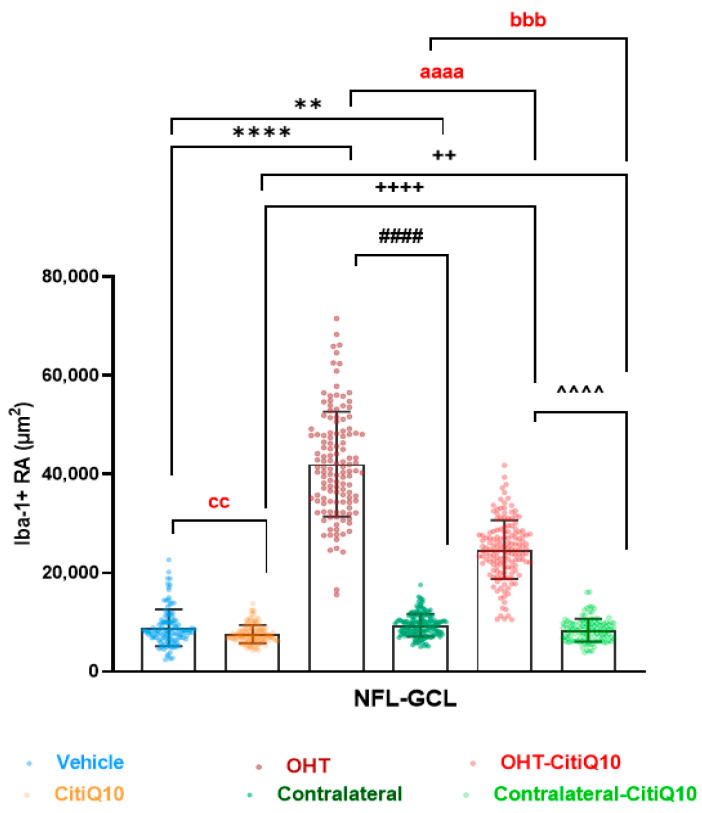
Retinal area occupied by Iba-1 + (Iba1-RA) in untreated and Citicoline + CoQ10-treated eyes, 3 days after ocular hypertension (OHT) induction in the nerve fiber layer–ganglion cell layer (NFL-GCL). Data are median with interquartile range; each data point in the column graph denotes an individual measure of the Iba1-RA per area of 0.1502 mm^2^. Statistical significance indicators: ** *p* < 0.01, **** *p* < 0.0001; vs. vehicle. ++ *p* < 0.01, ++++ *p* < 0.0001; vs. CitiQ10. #### *p* < 0.0001; OHT vs. contralateral. ^^^^ *p* < 0.0001; OHT-CitiQ10 vs. contralateral-CitiQ10. aaaa *p* < 0.0001; OHT vs. OHT-CitiQ10. bbb *p* < 0.001; contralateral vs. contralateral-CitiQ10. cc *p* < 0.01; vehicle vs. CitiQ10.

In relation to Iba1-RA **in the nerve fiber layer–ganglion cell layer (NFL-GCL)**, both untreated OHT eyes and those treated with Citicoline + CoQ10 showed an increase in Iba1-RA compared to their respective controls (vehicle and CitiQ10) and contralateral eyes (*p* < 0.0001, in all cases). Contralateral eyes, both treated and untreated, also showed a significant increase in Iba1-RA compared to their controls (vehicle and CitiQ10) (*p* < 0.01, in all cases). When comparing Citicoline + CoQ10-treated retinas with untreated ones, we found a significant decrease in Iba1-RA among controls (CitiQ10 vs. vehicle; *p* < 0.01), contralateral eyes (*p* < 0.001), and OHT eyes (*p* < 0.0001) (Figure 4).

##### Arbor Area

**Figure 5:**
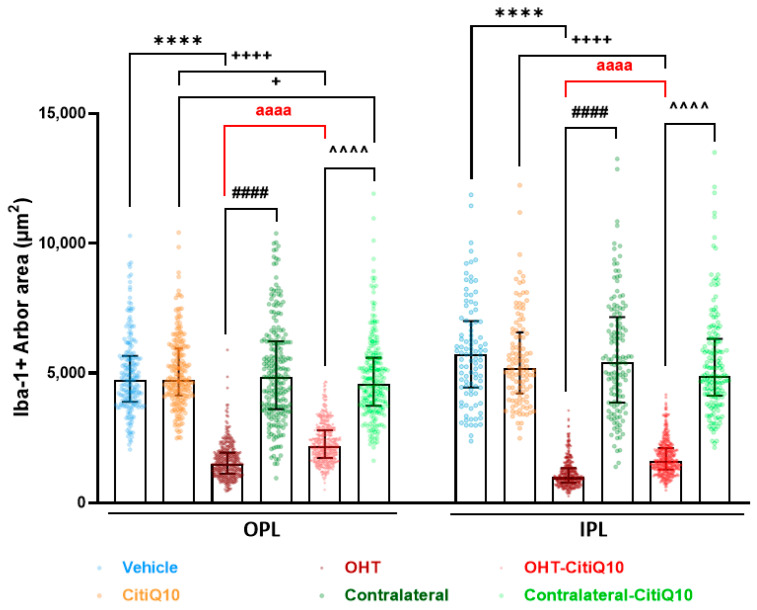
Comparison of the arbor area of Iba-1 + cells in untreated and Citicoline + CoQ10-treated eyes, 3 days after ocular hypertension (OHT) induction in the outer plexiform layer (OPL) and inner plexiform layer (IPL). Data are median with interquartile range; each data point in the column graph denotes an individual measure of the arbor area of Iba-1+ cells. Statistical significance indicators: **** *p* < 0.0001; vs. vehicle. + *p* < 0.05, ++++ *p* < 0.0001; vs. CitiQ10. #### *p* < 0.0001; OHT vs. contralateral. ^^^^ *p* < 0.0001; OHT-CitiQ10 vs. contralateral-CitiQ10. aaaa *p* < 0.0001; OHT vs. OHT-CitiQ10.

In the OPL and IPL, a reduction in the arbor area, indicative of microglial process retraction, was observed in both OHT eyes (untreated and treated with Citicoline + CoQ10) compared to their controls (vehicle and CitiQ10) and contralateral eyes (*p* < 0.0001, in all cases). In Citicoline + CoQ10-treated eyes compared to untreated ones, an increase in arbor area (less process retraction) was observed in OHT eyes (*p* < 0.0001, in all cases). However, in the OPL of contralateral-CitiQ10, a decrease in arbor area was found compared to their control (CitiQ10) (Figure 5).

##### Cell Body Area

**Figure 6:**
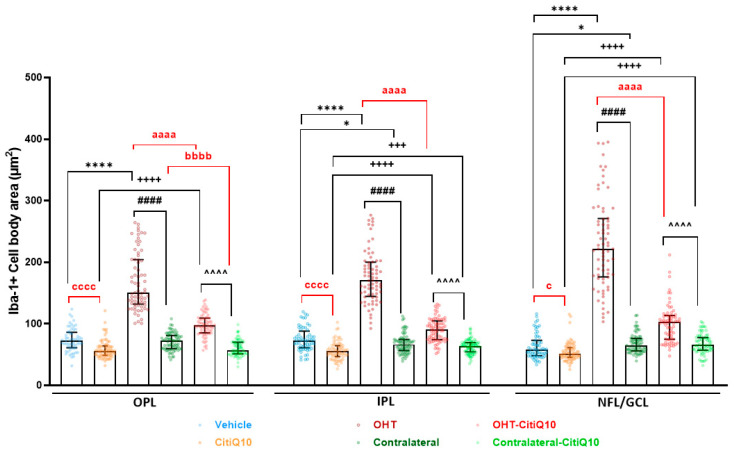
Cell body area of Iba-1 + cells in untreated and Citicoline + CoQ10-treated eyes, 3 days after ocular hypertension (OHT) induction in the outer plexiform layer (OPL), inner plexiform layer (IPL), and nerve fiber layer–ganglion cell layer (NFL-GCL). Data are median with interquartile range; each data point in the column graph denotes an individual measure of the cell body area of Iba-1+ cells. Statistical significance indicators: * *p* < 0.05, **** *p* < 0.0001; vs. vehicle. +++ *p* < 0.001, ++++ *p* < 0.0001; vs. CitiQ10. #### *p* < 0.0001; OHT vs. contralateral. ^^^^ *p* < 0.0001; OHT-CitiQ10 vs. contralateral-CitiQ10. aaaa *p* < 0.0001; OHT vs. OHT-CitiQ10. bbbb *p* < 0.0001; contralateral vs. contralateral-CitiQ10. c *p* < 0.05, cccc *p* < 0.0001; vehicle vs. CitiQ10.

In the **OPL, IPL, and NFL-GCL**, an increase in cell body area was found in both untreated and Citicoline + CoQ10-treated OHT eyes compared to their respective controls (vehicle and CitiQ10) and contralateral eyes (*p* < 0.0001, in all cases). In untreated contralateral eyes, a slight decrease in cell body area was found in the IPL (*p* < 0.05) and an increase in the NFL-GCL (*p* < 0.05) compared to their vehicle. In Citicoline + CoQ10-treated eyes compared to untreated ones, a significant decrease in cell body area was found among controls (CitiQ10 vs. vehicle) (*p* < 0.0001 in IPL and OPL and, *p* < 0.05 in NFL-GCL), among contralateral eyes (*p* < 0.001 only in the OPL), and among OHT eyes (*p* < 0.0001 in OPL, IPL, and NFL-GCL). An increase in cell body area was also observed in contralateral-CitiQ10 compared to their control (CitiQ10) (*p* < 0.001 in the IPL, and *p* < 0.0001 in NFL-GCL) (Figure 6).

##### Number of Vertical Processes Connecting the OPL and OS

**Figure 7:**
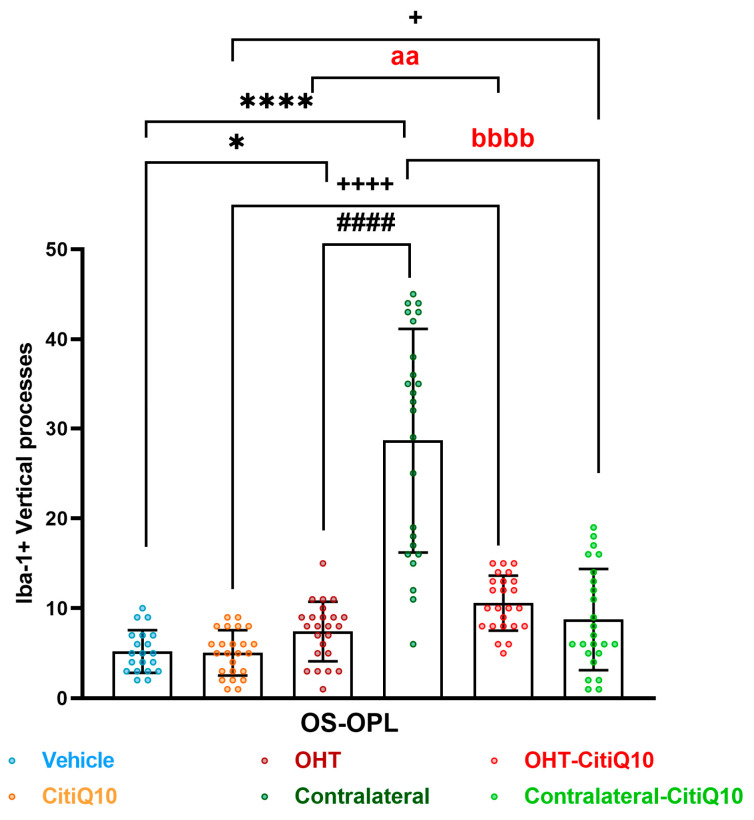
Comparison of Iba-1 + vertical processes (VP) in untreated and Citicoline + CoQ10-treated eyes, 3 days after ocular hypertension (OHT) induction connecting the photoreceptor outer segment layer (OS) and the outer plexiform layer (OPL). Data are median with interquartile range; each data point in the column graph denotes an individual measure of the of Iba-1+ VP. Statistical significance indicators: * *p* < 0.05, **** *p* < 0.0001; vs. vehicle. + *p* < 0.05, ++++ *p* < 0.0001; vs. CitiQ10. #### *p* < 0.0001; OHT vs. contralateral. aa *p* < 0.01; OHT vs. OHT-CitiQ10. bbbb *p* < 0.0001; contralateral vs. contralateral-CitiQ10.

The Citicoline + CoQ10-treated and untreated OHT eyes exhibited a significant increase in the number of vertical processes compared to their respective controls (vehicle and CitiQ10) (*p* < 0.05 for untreated eyes, and *p* < 0.0001 for treated eyes). Similarly, this increase was observed in the contralateral eyes, both treated and untreated, compared to their controls (vehicle and CitiQ10) (*p* < 0.0001 for untreated eyes, and *p* < 0.05 for treated eyes), and in the untreated contralateral eyes compared to their OHT counterparts (*p* < 0.0001). In the OHT eyes treated with Citicoline + CoQ10, there was a significant increase in vertical processes compared to untreated OHT eyes (*p* < 0.01). Conversely, in the Contralateral-CitiQ10, there was a significant decrease in vertical processes compared to their untreated contralateral counterparts (*p* < 0.0001) (Figure 7).

#### 2.2.2. The Combination of Citicoline and CoQ10 Reduces the Down-Regulation of Microglial P2RY12 Expression in OHT Eyes

**Figure 8:**
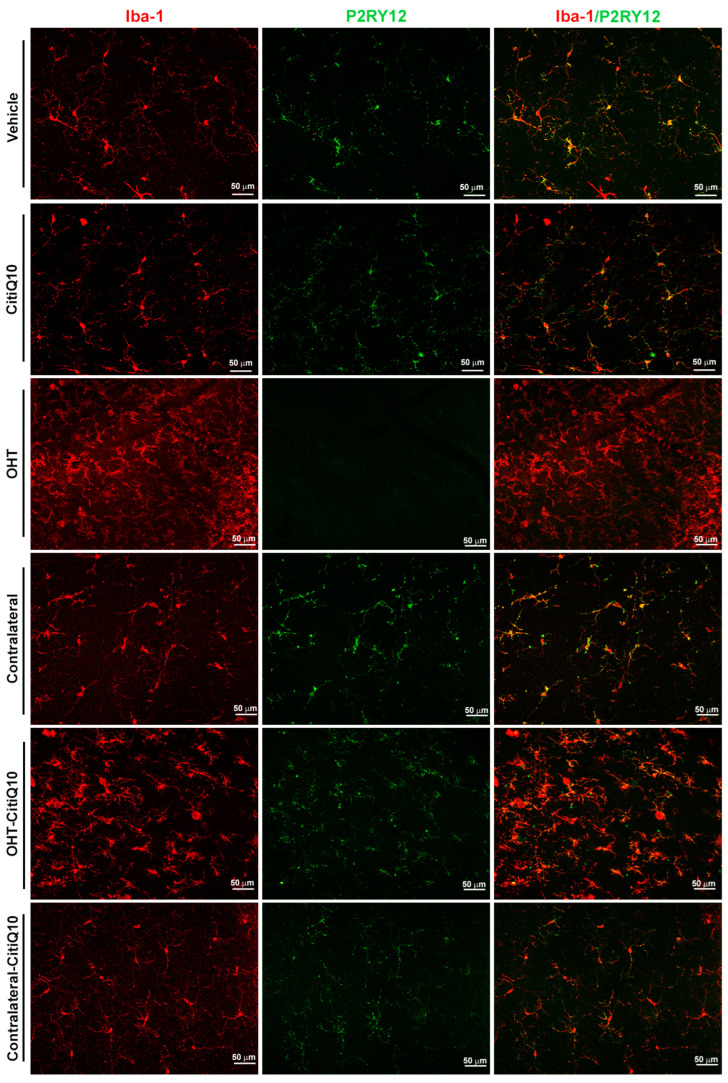
P2RY12 expression in the nerve fiber layer–ganglion cell layer (NFL-GCL) in untreated and Citicoline + CoQ10-treated eyes, 3 days after ocular hypertension (OHT) induction. The images show retinal whole-mounts with double immunostaining against Iba-1 and P2RY12, as well as when merged.

**Figure 9:**
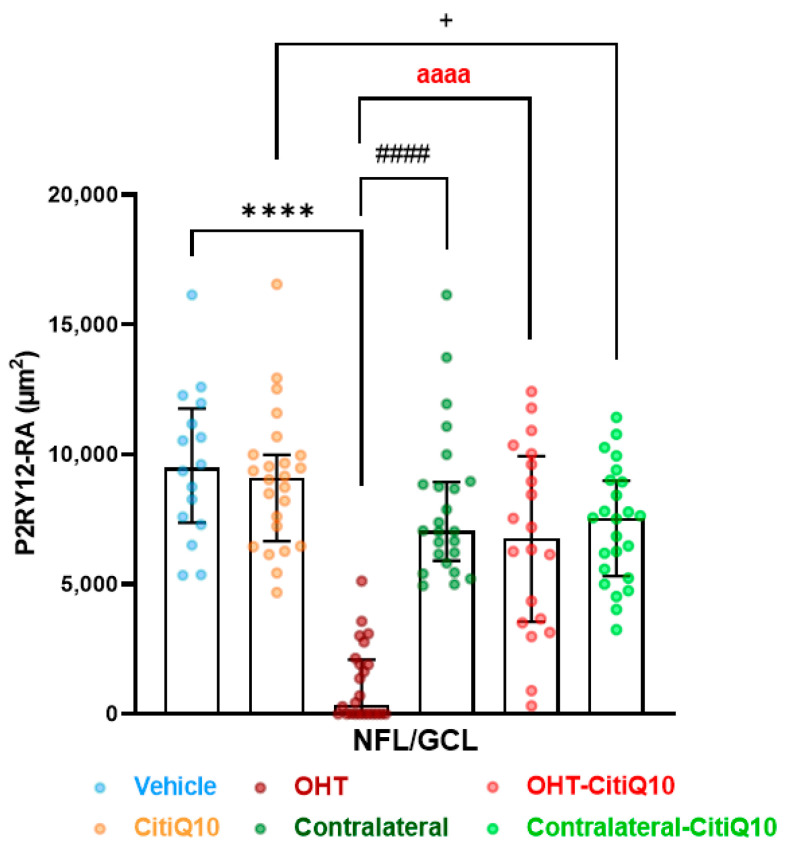
Retinal area occupied by P2RY12+ expression (P2RY12-RA) in untreated and Citicoline + CoQ10-treated eyes, 3 days after ocular hypertension (OHT) induction in the nerve fiber layer–ganglion cell layer (NFL-GCL). Data are median with interquartile range; each data point in the column graph denotes an individual measure of the P2RY12-RA per area of 0.1502 mm^2^. Statistical significance indicators: **** *p* < 0.0001; vs. vehicle. + *p* < 0.05; vs. CitiQ10. #### *p* < 0.0001; OHT vs. contralateral. aaaa *p* < 0.0001; OHT vs. OHT-CitiQ10.

In both vehicle and CitiQ10 animals, all Iba-1+ cells, excluding perivascular microglia and dendritic cells in the juxtapapillary area and peripheral retina, expressed P2RY12. Following laser induction in untreated OHT eyes, P2RY12 expression in Iba-1+ cells was significantly down-regulated compared to the vehicle and contralateral eyes (*p* < 0.0001, for both). Treatment with Citicoline + CoQ10 resulted in a smaller decrease in P2RY12 expression in OHT-CitiQ10 eyes (*p* < 0.0001). The contralateral eyes of untreated animals expressed P2RY12 at a level similar to the control (vehicle), whereas the contralateral-CitiQ10 eyes showed a slight decrease in P2RY12 expression compared to their control (CitiQ10) (*p* < 0.05) (Figure 8 and Figure 9).

#### 2.2.3. Macroglial Cells

**Figure 10:**
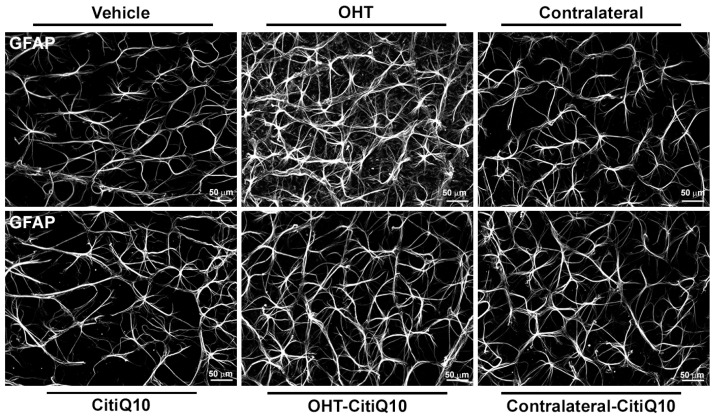
Comparison of GFAP+ cells in untreated and Citicoline + CoQ10-treated eyes in the nerve fiber layer–ganglion cell layer (NFL-GCL), 3 days after ocular hypertension (OHT) induction. The images show retinal whole-mounts with GFAP immunostaining.

**Figure 11:**
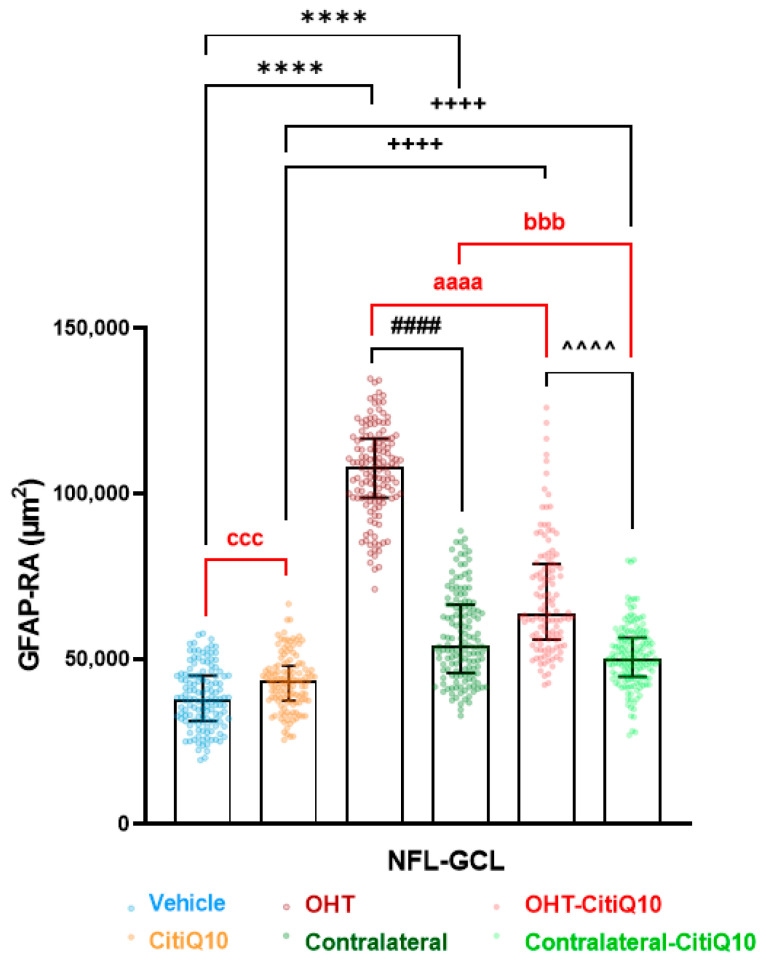
Retinal area occupied by GFAP+ expression (GFAP-RA) in untreated and Citicoline + CoQ10-treated eyes, 3 days after ocular hypertension (OHT) induction in the nerve fiber layer–ganglion cell layer (NFL-GCL). Data are median with interquartile range; each data point in the column graph denotes an individual measure of the GFAP-RA per area of 0.1502 mm^2^. Statistical significance indicators: **** *p* < 0.0001; vs. vehicle. ++++ *p* < 0.0001; vs. CitiQ10. #### *p* < 0.0001; OHT vs. contralateral. ^^^^ *p* < 0.0001; OHT-CitiQ10 vs. contralateral-CitiQ10. aaaa *p* < 0.0001; OHT vs. OHT-CitiQ10. bbb *p* < 0.001; contralateral vs. contralateral-CitiQ10. ccc *p* < 0.001; vehicle vs. CitiQ10.

In the **NFL-GCL**, both untreated OHT eyes and those treated with Citicoline + CoQ10 exhibited an increase in GFAP-RA compared to their respective controls (vehicle and CitiQ10) and contralateral eyes (*p* < 0.0001, in all cases). Contralateral eyes, whether treated or untreated, also showed a significant increase in GFAP-RA compared to their controls (vehicle and CitiQ10) (*p* < 0.0001, in all cases). When comparing Citicoline + CoQ10-treated retinas to untreated ones, there was a significant decrease in GFAP-RA in both contralateral-CitiQ10 eyes (*p* < 0.001) and OHT-CitiQ10 eyes (*p* < 0.0001). Additionally, comparing the controls revealed a significant increase in GFAP-RA in CitiQ10 compared to the vehicle (*p* < 0.001) (Figure 10 and Figure 11).

### 2.3. Nuclei of the Visual Pathway: The Combination of Citicoline and CoQ10 Reduces Glial Activation Produced by Laser-Induced OHT

#### 2.3.1. Dorsolateral Geniculate Nucleus (dLGN)

##### Iba-1+ Cells

**Figure 12:**
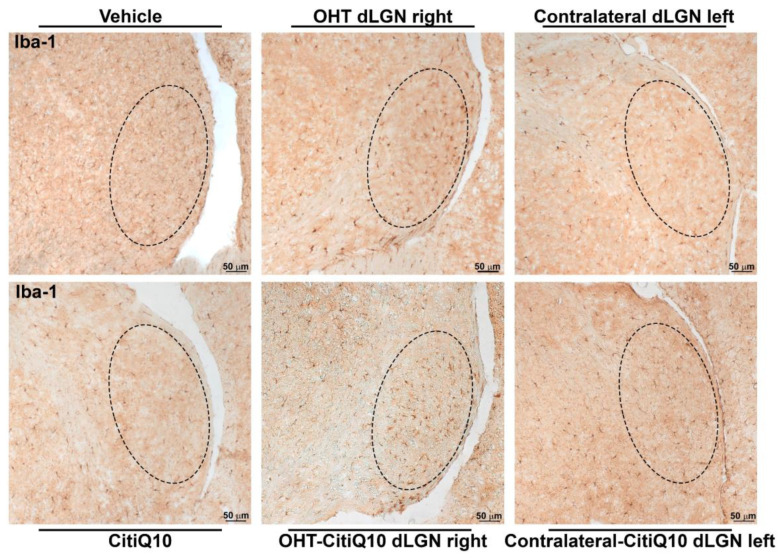
Comparison of Iba-1+ cells in untreated and Citicoline + CoQ10-treated eyes in the dorsolateral geniculate nucleus (dLGN), 7 days after ocular hypertension (OHT) induction. The images show brain sections with Iba-1 immunostaining.

**Figure 13:**
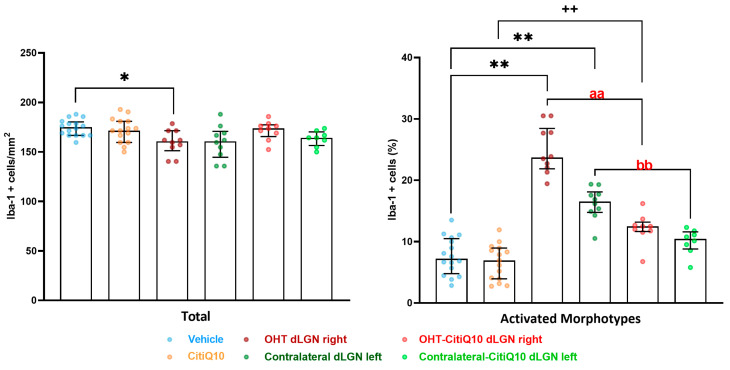
Analysis of Iba1+ cells (number of Iba-1+ cells (left) and % of activated morphotypes of Iba-1+ cells (right)) in untreated and Citicoline + CoQ10-treated eyes, 7 days after ocular hypertension (OHT) induction in the dorsolateral geniculate nucleus (dLGN). Data are median with interquartile range; each data point in the column graph denotes an individual measure of the Iba-1+ cells (count; % count). Statistical significance indicators: * *p* < 0.05, ** *p* < 0.01; vs. vehicle. ++ *p* < 0.01; vs. CitiQ10. aa *p* < 0.01; OHT dLGN right vs. OHT-CitiQ10 dLGN right. bb *p* < 0.01; contralateral dLGN left vs. contralateral-CitiQ10 dLGN left.

Regarding microglial study, the dLGN displayed a significant lower number of Iba1+ cells in the OHT dLGN right when compared to the vehicle (*p* < 0.05). However, a higher percentage of activated morphotypes of Iba1+ cells was found in both the OHT dLGN right and the OHT-CitiQ10 right (*p* < 0.01) compared to their respective controls (vehicle and CitiQ10) (*p* < 0.01, in both), and in the contralateral dLGN left (*p* < 0.01) in comparison to the vehicle. When comparing Citicoline + CoQ10-treated dLGN with untreated ones, the percentage of activated morphotypes of Iba1+ cells was lower (*p* < 0.01) in OHT-CitiQ10 dLGN right and in contralateal-CitiQ10 dLGN left (Figure 12 and Figure 13).

##### GFAP+ Cells

**Figure 14:**
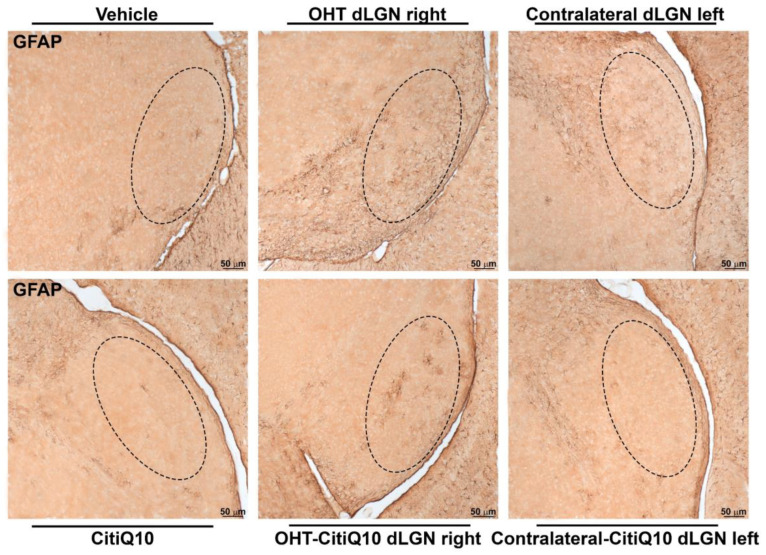
Comparison of GFAP+ cells in untreated and Citicoline + CoQ10-treated eyes in the dorsolateral geniculate nucleus (dLGN), 7 days after ocular hypertension (OHT) induction. The images show brain sections with GFAP immunostaining.

**Figure 15:**
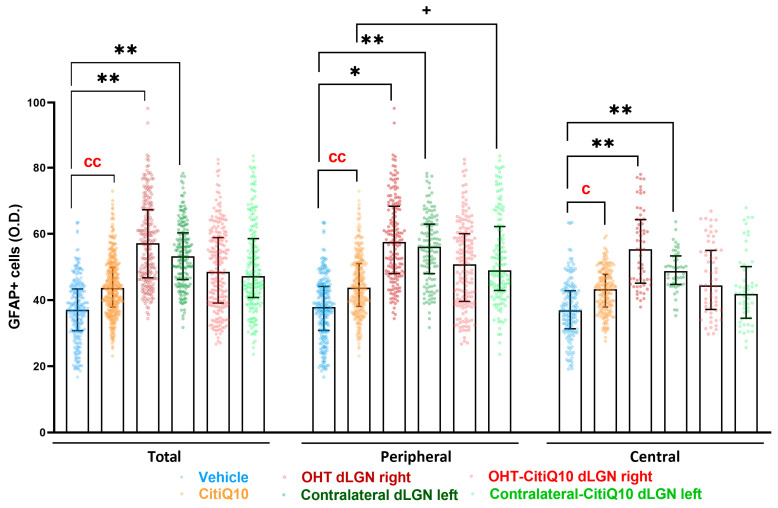
Analysis of GFAP+ cells (O.D.) (as a whole, and peripheral zone and central zone) in untreated and Citicoline + CoQ10-treated eyes, 7 days after ocular hypertension (OHT) induction in the dorsolateral geniculate nucleus (dLGN). Data are median with interquartile range; each data point in the column graph denotes an individual measure of the GFAP+ cells (O.D.). Statistical significance indicators: * *p* < 0.05, ** *p* < 0.01; vs. vehicle. + *p* < 0.05; vs. CitiQ10. c *p* < 0.05, cc *p* < 0.01; vehicle vs. CitiQ10. O.D.: optical density.

For the macroglial study, when analyzing the dLGN as a whole, the OHT dLGN right showed a significant increase in GFAP expression compared to the vehicle (*p* < 0.01); similar results were obtained when analyzing the peripheral zone of the dLGN (*p* < 0.05) and the central zone of the dLGN (*p* < 0.01). Moreover, a significant increase in GFAP expression in the contralateral left dLGN compared to the vehicle was found in the total (*p* < 0.01), peripheral (*p* < 0.01), and central (*p* < 0.01) dLGN. Only in the peripheral zone was a significant increase in GFAP expression in the contralateral-CitiQ10 dLGN left (*p* < 0.05) found compared to CitiQ10. In Citicoline + CoQ10-treated eyes compared to untreated ones, a significant increase in GFAP expression was found among controls (CitiQ10 vs. vehicle) (*p* < 0.01, in total and peripheral; *p* < 0.05, in central) (Figure 14 and Figure 15).

#### 2.3.2. Superior Colliculus (SC)

##### Iba-1+ Cells

**Figure 16:**
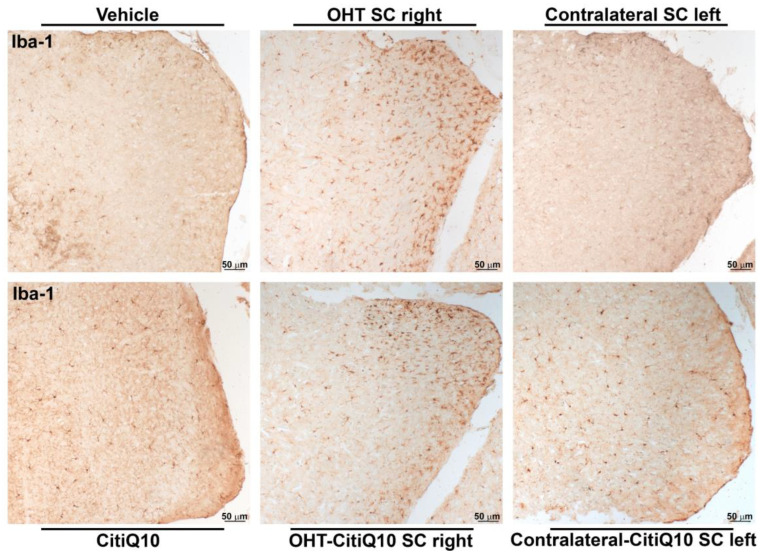
Comparison of Iba-1+ cells in untreated and Citicoline + CoQ10-treated eyes in the superior colliculus (SC), 7 days after ocular hypertension (OHT) induction. The images show brain sections with Iba-1 immunostaining.

**Figure 17:**
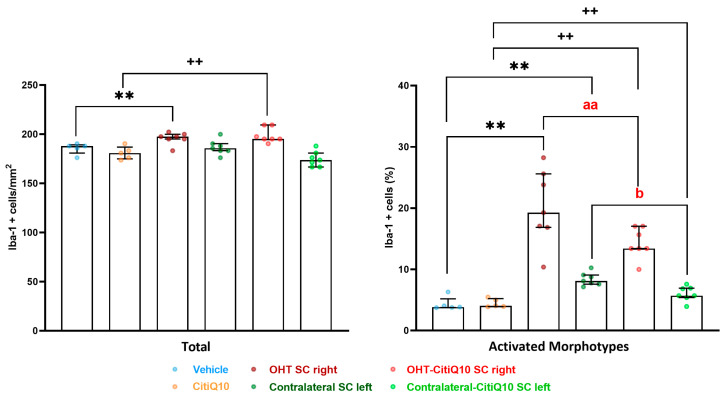
Analysis of Iba1+ cells (number of Iba-1+ cells (left) and % of activated morphotypes of Iba-1+ cells (right)) in untreated and Citicoline + CoQ10-treated eyes, 7 days after ocular hypertension (OHT) induction in the superior colliculus (SC). Data are median with interquartile range; each data point in the column graph denotes an individual measure of the Iba-1+ cells (count; % count). Statistical significance indicators: ** *p* < 0.01; vs. vehicle. ++ *p* < 0.01; vs. CitiQ10. aa *p* < 0.01; OHT SC right vs. OHT-CitiQ10 SC right. b *p* < 0.05; contralateral SC left vs. contralateral-CitiQ10 SC left.

The SC showed a higher number of Iba1+ cells in the OHT SC right and the OHT-CitiQ10 SC right compared to their respective controls (vehicle and CitiQ10) (*p* < 0.01). Furthermore, the percentage of activated morphotypes of Iba1+ cells was also higher in the OHT SC right and the OHT-CitiQ10 SC right compared to their respective controls (vehicle and CitiQ10) (*p* < 0.01). Similarly, the contralateral groups (contralateral SC left and contralateral-CitiQ10 SC left) also showed a higher percentage of activated Iba1+ cells compared to their controls (vehicle and CitiQ10) (*p* < 0.01). In regard to the Citicoline + CoQ10 treatment, the percentage of activated morphotypes of Iba1+ cells was lower in the OHT-CitiQ10 SC right (*p* < 0.01) and contralateral-CitiQ10 SC left (*p* < 0.05) compared to the OHT SC right and contralateral SC left, respectively (Figure 16 and Figure 17).

##### GFAP+ Cells

**Figure 18:**
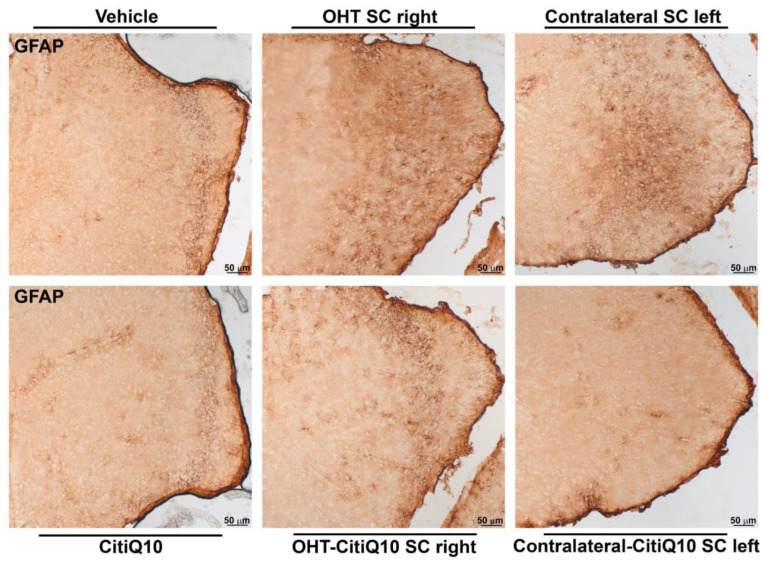
Comparison of GFAP+ cells in untreated and Citicoline + CoQ10-treated eyes in the superior colliculus (SC), 7 days after ocular hypertension (OHT) induction. The images show brain sections with GFAP immunostaining.

**Figure 19:**
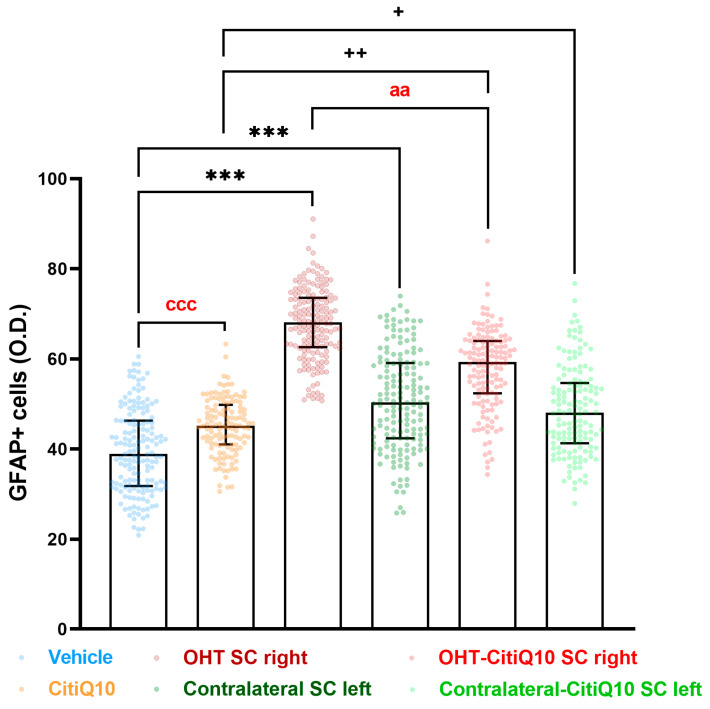
Analysis of GFAP+ cells (O.D.) in untreated and Citicoline + CoQ10-treated eyes, 7 days after ocular hypertension (OHT) induction in the superior colliculus (SC). Data are median with interquartile range; each data point in the column graph denotes an individual measure of the GFAP+ cells (O.D.). Statistical significance indicators: *** *p* < 0.001; vs. vehicle. + *p* < 0.05, ++ *p* < 0.01; vs. CitiQ10. aa *p* < 0.01; OHT SC right vs. OHT-CitiQ10 SC right ccc *p* < 0.001; vehicle vs. CitiQ10. O.D.: optical density.

In the superior colliculus (SC), both untreated OHT SC right and those treated with Citicoline + CoQ10 showed increased GFAP expression compared to their respective controls (vehicle and CitiQ10) (*p* < 0.001 for OHT SC right; *p* < 0.01 for OHT-CitiQ10 SC right). The contralateral SC left, both treated and untreated, also showed significantly higher GFAP expression compared to their controls (vehicle and CitiQ10) (*p* < 0.001 for contralateral SC left; *p* < 0.05 for contralateral-CitiQ10 SC left). When comparing Citicoline + CoQ10-treated SC with untreated ones, we found a significant decrease in GFAP expression in OHT-CitiQ10 SC right (*p* < 0.01). When comparing the controls, we found an increase in GFAP expression in CitiQ10 compared to the vehicle (*p* < 0.001) (Figure 18 and Figure 19).

#### 2.3.3. Visual Cortex (V1)

##### Iba-1+ Cells

**Figure 20:**
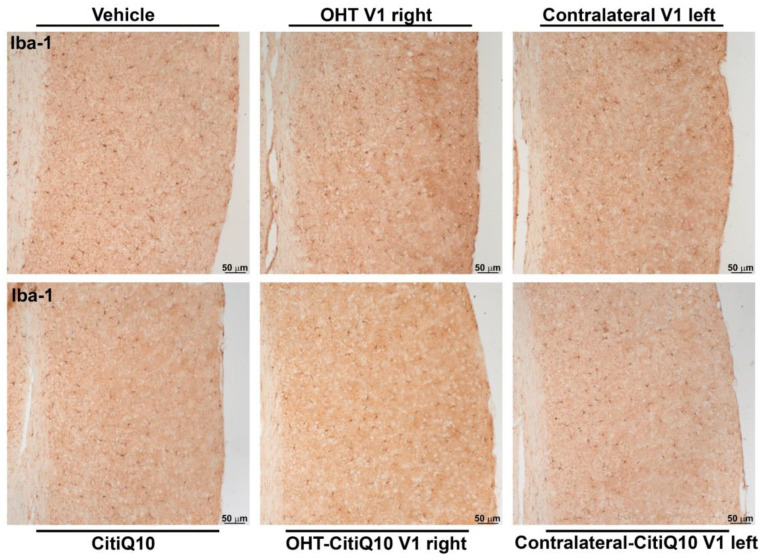
Comparison of Iba-1+ cells in untreated and Citicoline + CoQ10-treated eyes in the visual cortex (V1), 7 days after ocular hypertension (OHT) induction. The images show brain sections with Iba-1 immunostaining.

**Figure 21:**
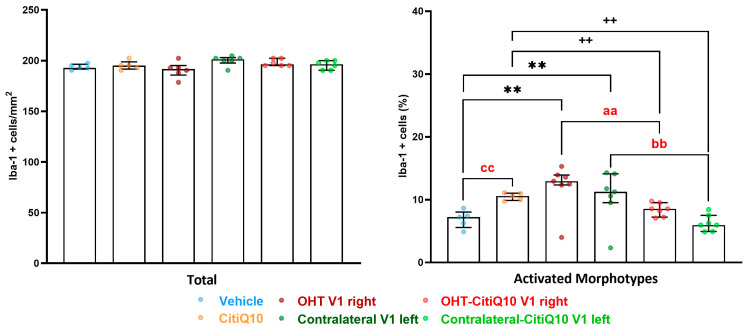
Analysis of Iba1+ cells (number of Iba-1+ cells (left) and % of activated morphotypes of Iba-1+ cells (right)) in untreated and Citicoline + CoQ10-treated eyes, 7 days after ocular hypertension (OHT) induction in the visual cortex (V1). Data are median with interquartile range; each data point in the column graph denotes an individual measure of the Iba-1+ cells (count; % count). Statistical significance indicators: ** *p* < 0.01; vs. vehicle. ++ *p* < 0.01; vs. CitiQ10. aa *p* < 0.01; OHT V1 right vs. OHT-CitiQ10 V1 right. bb *p* < 0.01; contralateral V1 left vs. contralateral-CitiQ10 V1 left. cc *p* < 0.01; vehicle vs. CitiQ10.

When analyzing the activated morphotypes of Iba1+ cells, both the OHT and contralateral V1 groups showed a higher percentage of activated cells compared to their respective controls (vehicle and CitiQ10) (*p* < 0.01, in all cases). However, the Citicoline + CoQ10 treatment resulted in a lower percentage of activated Iba1+ cells in the OHT-CitiQ10 V1 right and contralateral-CitiQ10 V1 left compared to the OHT SC right and contralateral SC left, respectively (*p* < 0.01, in both cases). Comparing the controls, we found an increase in the percentage of activated Iba1+ cells in CitiQ10 compared to the vehicle (*p* < 0.01) (Figure 20 and Figure 21).

##### GFAP+ Cells

**Figure 22:**
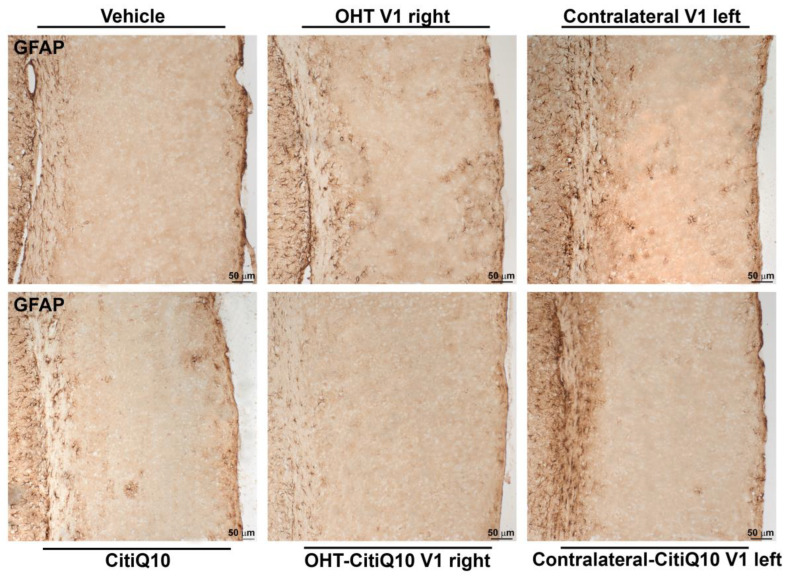
Comparison of GFAP+ cells in untreated and Citicoline + CoQ10-treated eyes in the visual cortex (V1), 7 days after ocular hypertension (OHT) induction. The images show brain sections with GFAP immunostaining.

**Figure 23:**
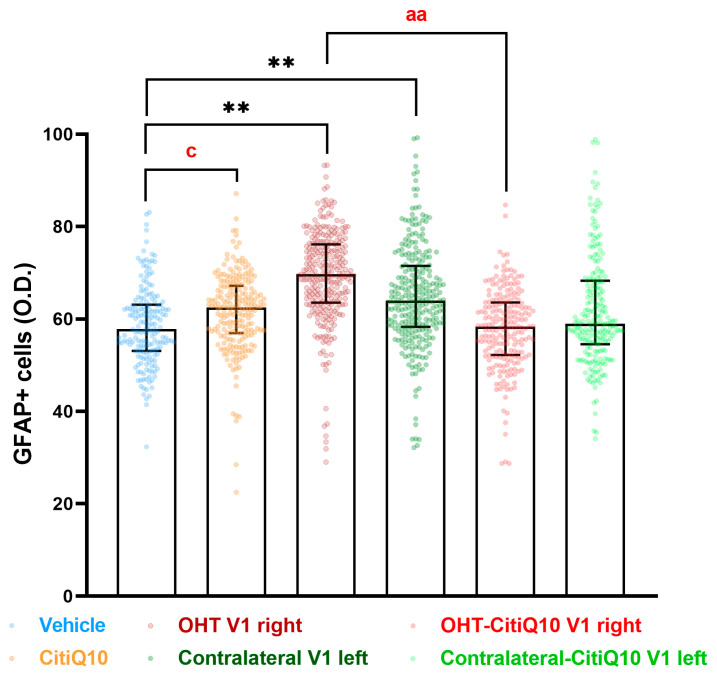
Analysis of GFAP+ cells (O.D.) in untreated and Citicoline + CoQ10-treated eyes, 7 days after ocular hypertension (OHT) induction in the visual cortex (V1). Data are median with interquartile range; each data point in the column graph denotes an individual measure of the GFAP+ cells (O.D.). Statistical significance indicators: ** *p* < 0.01; vs. vehicle. aa *p* < 0.01; OHT V1 right vs. OHT-CitiQ10 V1 right c *p* < 0.05; vehicle vs. CitiQ10. O.D.: optical density.

In the visual cortex (V1), a significant increment of GFAP expression was found in both the OHT V1 right (*p* < 0.01) and contralateral V1 left (*p* < 0.01) when compared to the vehicle. Furthermore, the Citicoline + Coenzyme Q10 combination showed a decrease in GFAP expression in the OHT-CitiQ10 V1 right compared to the OHT V1 right (*p* < 0.01). The comparison between controls showed an increase in the percentage of activated Iba1+ cells in the CitiQ10 group compared to the vehicle group (*p* < 0.05) (Figure 22 and Figure 23).

## 3. Discussion

This study is the first to show that the combination of Citicoline and CoQ10 controls glial activation, potentially exerting an anti-inflammatory effect in the retina and along the visual pathway in an experimental model of ocular hypertension. However, it is a preliminary study that analyzes the morphological markers of microglial and macroglial activation. Only two previous studies have analyzed the benefits of combining Citicoline and CoQ10. One study used rat astrocyte cultures exposed to oxidative stress [32], and the other, conducted by our group in the same experimental model used here, demonstrated neuroprotective effects [7]. In the latter study, it was demonstrated that the combination of Citicoline + CoQ10 may exert a neuroprotective effect on RGCs and in the dLGN of the visual pathway in a laser-induced OHT model in mice. However, the study did not analyze whether this compound could be controlling the inflammatory process to promote the survival of RGCs.

In this study, we used a unilateral laser-induced hypertension model to temporarily raise the IOP by altering aqueous humor drainage in adult CD1 Swiss albino male mice. This procedure causes significant and sectorial death of RGCs [33,34,35] and glial cell activation [15,18,23], similar to changes seen in human glaucomatous neuropathy [19]. This model is useful for evaluating the neuroprotective and anti-inflammatory effects of various substances [34,36].

In our study, the combination of Citicoline and CoQ10 showed a hypotensive effect at 24 h and 3 days after laser treatment. At these times, a significant inflammatory process occurs, with increased IL-6 levels associated with elevated IOP [37,38], and typical glial activation changes [18,23,36,39]. In rat astrocyte cultures exposed to oxidative stress, this combination reduced the expression of inflammatory proteins (NF-κB, TNFα) [32]. This suggests that the combination may offer enhanced protection against early inflammation, resulting in lower IOP peaks.

Chronic neuroinflammation is a complex process involving the persistent activation of immune cells in the central nervous system (CNS), mainly microglia and astrocytes, in response to various pathological stimuli, such as increased IOP. This sustained inflammatory response is linked to a wide range of disorders, including neurodegenerative diseases like glaucoma [14]. Increased IOP triggers microglial activation in humans [19] and in experimental glaucoma models [15,20,40,41,42]. In a previous study [23], we analyzed glial cell activation over time after inducing OHT in mice and found the most notable changes at 3 days post induction. Thus, we chose this time point to study the anti-inflammatory effects of Citicoline and CoQ10. Although 3 days may seem short, it is significant due to the shorter lifespan of mice (1.5–3 years) [43,44], equating to about 1.15 human years [44].

Microglial activation involves changes in cell shape, an increased cell number, and the release of inflammatory molecules like cytokines, chemokines, and ROS [45]. While short-term activation can be protective, long-term activation is harmful [46]. Activated microglia release substances such as IL-6, TNF-α, and IL-1β, which can damage neurons and affect synaptic plasticity [47]. This chronic inflammation creates a feedback loop, where cytokines further activate microglia, perpetuating inflammation and tissue damage [48]. Astrocytes also play a role, either protecting neurons or perpetuating inflammation [49]. Astrocytes, in the retina, maintain the blood–retinal barrier (BRB), but chronic neuroinflammation can disrupt the BRB, allowing immune cells to enter the CNS and worsening neurodegeneration [14]. In our mouse model of OHT, microglia respond to increased IOP by releasing IL-6, which activates astrocytes to release VEGF, BDNF, TNF-α, and IL-1β, modulating inflammation [36,39].

As in previous studies [23], we found that 3 days after OHT induction, retinal microglia showed characteristic activation changes in OHT eyes, such as an increase in the number of microglia, process retraction (decrease in arbor area), an increase in soma size, an increase in vertical processes, and a decrease in the expression of the P2RY12 receptor, a sensitive indicator of the shift from resting to activated microglia. Also, as in a previous study [22], we observed an increase in the retinal area occupied by GFAP, a sensitive marker for astroglia and Müller glial cell activation [50,51,52,53]. These morphological glial changes are linked to molecular changes, such as the up-regulation of pro-inflammatory cytokines like IFN-γ and IL-6 [23,36,39], cytokines capable of triggering the production of nitric oxide and ROS, resulting in neuronal death [54,55,56]. This aligns with our previous observations, in which a decrease in the expression of the RGC marker, Brn3a, was reported 3 days after OHT induction, with a remarkable RGC death 5 and 7 days after laser induction [7,34,39]. The relationship between microglial activation and RGC death has been observed not only in experimental glaucoma models but also in human glaucoma [19].

The microglial and macroglial activation changes were also observed in the retinas of the contralateral eyes, although more subtly, such as increased Iba-RA and soma size, without a decrease in P2RY12 receptor regulation [23], and an increase in the retinal area occupied by GFAP [22]. Additionally, a previous study found that these morphological changes corresponded with molecular changes like the up-regulation of IL-1β, IL-6, and IL-10 [36,39]. This indicates that the increase in IOP in the OHT eye induces retinal microglial and astroglial changes in the contralateral eyes that are not related to RGC death in these eyes [39]. Several theories may explain this glial activation in the contralateral eye, including those related to hematic pathways, transmission through the chiasm, and the involvement of the immune system [57,58].

Treatment with citicoline and CoQ10 led to a reduction in glial activation changes in both microglial and macroglial cells, observed in OHT eyes and their contralateral counterparts, compared to untreated eyes. In OHT-CitiQ10 retinas, we found (i) fewer microglial cells in the OS, OPL, and IPL; (ii) reduced Iba-RA in the NFL-GCL, indicating fewer microglia in that layer; (iii) an increased arbor area in the OPL and IPL, showing less microglial process retraction; (iv) a decreased cell body area in the OPL, IPL, and NFL-GCL, indicating less soma enlargement; (v) increased vertical processes connecting the OS and OPL, showing microglia extending processes rather than moving amoeboid microglia; (vi) higher P2RY12 receptor expression, indicating reduced microglial activation; and (vii) lower GFAP-RA, indicating less astrocyte and Müller glia activation. The subtle changes in contralateral eyes were also diminished in CitiQ10-treated eyes, showing decreased GFAP-RA, lower Iba-RA in the NFL-GCL, a smaller microglial cell body area in the OPL, and fewer vertical processes connecting the OS and OPL. The reduced glial cell activation observed in the retinas of animals treated with Citicoline and Coenzyme Q10 suggests that this combination may control the inflammatory process associated with IOP increase. Consequently, it may help prevent RGC death, as observed in a parallel study analyzing the neuroprotective effect of this combination 7 days after OHT induction in this experimental model [7].

Surprisingly, we found higher GFAP-RA levels in retina and higher GFAP expression in the different nuclei of the visual pathway, in the controls that took Citicoline + CoQ10 compared to those who did not, and although this difference was small, it was statistically significant. Since in the previous study where we analyzed the neuroprotective effect of Citicoline + CoQ10, we only found neuronal death in the OHT eyes not treated with the combination [7], this activation does not seem to be associated with neurodegeneration. The explanation for this macroglial activation in the controls treated with the compound could be the fact that many drugs exert neuroprotective effects by activating glial cells, modulating their functions in such a way that they can induce direct neurotrophic effects (greater release of neurotrophic factors, increased energy supply, etc.) or trigger more subtle indirect changes that lead to an overall improvement in neuronal cell function (greater glutamate uptake, reorganization of metabolic pathways, modulation of synaptic transmission, etc.) [59,60,61]

Visual information processed by the retina is transmitted to various regions of the CNS, and in rodents, approximately 50 brain areas involved in the visual pathway have been identified [62]. Among these, the majority project to the SC, while a substantial number target the dLGN [63]. Subsequently, second-order neurons in these relay nuclei transmit information to V1 [63]. The study was performed 7 days after the OHT induction, because in a previous study, we observed that neurodegeneration begins to occur in the dLGN at this time point [7]; we thus hypothesized that the inflammatory process should have started by this time in these nuclei. The results obtained in the dLGN revealed that the OHT dLGN right exhibited a reduced number of Iba1+ cells compared to the vehicle group. However, a higher percentage of activated Iba1+ cell morphotypes was observed in both the right OHT dLGN and the contralateral left dLGN, with the right side showing a slightly greater increase than the left. In rodents, it is known that approximately 95% of RGC fibers decussate due to their laterally positioned eyes [63]; hence, any alteration in the left eye (OHT) would predominantly affect the right hemisphere, specifically the OHT dLGN right, which aligns with the findings of our study. The observed microglial activation may be attributed to the inflammatory processes previously described in the retina during glaucoma [16,23], and in the previous results described in this article, which propagate through the optic nerve, into the optic tract, and eventually reach the nuclei within the visual pathway [17].

Concerning macroglial activation, when the dLGN was analyzed in its entirety, the OHT dLGN right group exhibited a significant elevation in GFAP expression compared to the vehicle group. In rodents, the dLGN exhibits a functional subdivision determined by the afferent inputs, receiving approximately 5–10% of the RGC projections with an origin in the retina of the ipsilateral eye, a small central region in the dorsal part, while the remaining RGC afferents present an origin of the contralateral retina, and innervating the more peripheral region of the dLGN [64]. GFAP staining allowed us to distinguish these two areas, revealing a significant increase in GFAP expression in both the peripheral and central zones of the OHT dLGN right group. In a previous study conducted by our group [7], we demonstrated that, using the same model, the right OHT dLGN exhibited a decrease in the expression of the neuronal marker NeuN in both zones, although it was slightly more pronounced in the peripheral region, which receives the majority of RGC afferents from the OHT eye. This neurodegeneration coincides with the microglial and macroglial activation observed throughout the dLGN. We cannot rule out the possibility that the initial damage is somewhat greater in the peripheral zone, with the inflammatory process subsequently extending to the central region. Alternatively, the widespread microglial activation observed throughout the dLGN might, in turn, induce macroglial activation [65,66]. Furthermore, the contralateral left dLGN also exhibited a significant increase in GFAP expression when compared to the vehicle group, with this elevation observed across the total, peripheral, and central dLGN regions. Our group has previously demonstrated that, in this glaucoma model, damage can also be detected in the contralateral eye [16,22], which provides an explanation for the activation in the left hemisphere visual areas, likely mediated by the transmission of neuroinflammation through the optic tract; other researchers have observed that unilateral IOP elevation results in bilateral modifications in astrocytes within the retina and optic nerves [67,68]. The optic nerve is the link between neuroinflammation and neurodegeneration that occurs in the eye and the alterations that occur along the visual pathway. Two neurodegenerative events occur early in the optic nerve during glaucoma: the loss of anterograde transport in RGC axons [69,70] and alterations in astrocyte function and structure [71,72]. Cooper et al. (2025) [72] obtained similar results by using the microbead occlusion model to induce unilateral IOP elevation; they observed greater macroglial activation in both hemispheres in the SC and LGN one week after induction, although degeneration was induced only in one retina.

The SC displayed more Iba1+ cells in the OHT SC right and contralateral SC left, and the OHT SC right and contralateral SC left showed an increased percentage of Iba1+ activated cells. Moreover, GFAP expression was significantly increased in the OHT SC right, and the contralateral SC left also showed an increase, although it was less pronounced than that observed in the OHT SC right. As mentioned above, in mice, 85–90% of RGCs from the contralateral eye projected to the SC [73]. As stated above, glial activation in the right hemisphere can be explained by the damage in the OHT eye in this model of glaucoma; therefore, a higher activation of microglia and macroglia should be observed in the OHT SC right, as in our present results. Moreover, the dLGN and the SC are highly intertwined, with direct projections between them [74], and thus inflammation could also travel between these nuclei, besides the one originating from the retina. In our previous work, Matamoros et al. 2025 [7], we found no significant neuronal degeneration in the SC, though a high activation of glia seems to be occurring. The SC is a sensorimotor structure that integrates visual, auditory, and somatosensory information and contributes to the initiation of motor patterns [75,76]. Therefore, the partial loss of retinal inputs may not have been sufficient to produce detectable neuronal damage in the SC, as it continued to receive afferent inputs from other sensory pathways. Nevertheless, early functional alterations may already have been present, as suggested by the observed pro-inflammatory state, and could have become evident upon further analysis at later time points. As in the dLGN, the alterations observed in the contralateral left SC may be due to neuroinflammation transmitted from the OHT eye to the contralateral hemisphere through the optic tract.

Although in the V1, the analysis rendered no significant differences in the total number of Iba1+ cells, a higher percentage of Iba1+ activated cells was found in the OHT V1 right and contralateral V1 left, and an increase in the expression of GFAP-positive cells in both hemispheres. We found no significant neuronal degeneration in V1 in our previous study [7]. Similarly to what we have indicated in the SC, since V1 does not receive direct afferents from the retina but rather from other nuclei of the visual pathway [77], the absence of detectable neuronal loss may be due to the persistence of this indirect connection, though neuroinflammatory alterations transmitted from other nuclei can be observed. It is possible that, with studies conducted at later time points after OHT induction, more significant results might be detected in V1, both in terms of neurodegeneration and neuroinflammation.

The treatment with Citicoline and Coenzyme Q10 effectively reduced microglial activation in dLGN and microglial and macroglial activation in the SC and V1, both in OHT right and contralateral left. To the best of our knowledge, there are limited studies that thoroughly investigate the effects of Citicoline and Coenzyme Q10 on the visual pathway. Nevertheless, studies focusing exclusively on CoQ10 have demonstrated its anti-inflammatory properties in the CNS in the context of glaucoma [48,78]. Furthermore, CoQ10 has shown similar effects in other neuroinflammatory conditions, such as Alzheimer’s disease [79], and epilepsy [80], where the inhibition of microglial activation has been proposed as an underlying mechanism. The treatment with Citicoline and Coenzyme Q10 did not reverse the observed macroglial activation in dLGN. This finding supports the hypothesis that a complete reversal of microglial activation through treatment might be a prerequisite for achieving the reversal of macroglial activation. Furthermore, it is plausible that prolonging the treatment duration and/or increasing the dosage may increase the effects along the visual pathway, producing effects similar in magnitude to those observed in the retina. This is of particular relevance given that the progression of inflammation along the visual pathway is delayed, occurring later in time.

Few studies have investigated the anti-inflammatory properties of Citicoline and Coenzyme Q10 in experimental glaucoma models or related pathogenic mechanisms. However, all these studies highlight the anti-inflammatory capabilities of both compounds, as observed in this work. Only one study has analyzed the effect of the Citicoline–Coenzyme Q10 combination in rat astrocyte cultures under oxidative stress, and the administration of Citicoline or CoQ10 led to a decrease in the expression of inflammatory and pro-apoptotic proteins (TNFα, IL-6, NFkβ, CRLS1, SOD2, BAX, BCL-2). However, this reduction was more pronounced when both compounds were used together (Citicoline + CoQ10). The combination showed stronger synergistic effects in reducing oxidative stress, inflammation, and apoptosis, indicating a cumulative protective effect over time [32]. Other studies have used Citicoline or CoQ10 separately or combined with other compounds. In the genetic glaucoma model, in DBA/2J mice, 1% CoQ10 oral administration reduced GFAP expression, indicating its ability to reduce glial activation [28]. In an ischemia–reperfusion model in C57BL/6 mice, CoQ10 significantly reduced GFAP and Iba-1 expression, preventing the activation of astroglial and microglial cells in the ischemic retina through the blockade of oxidative stress [29]. In the same model in rats, MitoQ (a form of CoQ10) reduced inflammatory cytokine levels (IL-1β, IL-6, TNF-α) [30]. In pig retina explants in which degeneration was induced by H_2_O_2_ administration, CoQ10 significantly reduced the number of microglial cells and IL-8, suggesting an anti-inflammatory effect [81]. Idebenone, a synthetic version of CoQ10, showed neuroprotective effects in human astrocyte cultures under oxidative stress [82]. Finally, the combination of CoQ10 and vitamin E in a rat model of mechanical optic nerve damage decreased the number of GFAP+ astroglial cells and Iba-1+ microglial cells, as well as GFAP and Iba-1 expression [31]. In kainate-treated rats, where treatment activates ionotropic glutamate receptors causing excitotoxicity and neuronal death [83], Citicoline reduced pERK expression in the INL, IPL, and GCL, particularly in Müller cell bodies and astrocyte processes. This pathway regulates the production of inflammatory cytokines and iNOS expression in activated microglia [84]. In neuronal cells exposed to oxidative stress, Citicoline and CoQ10 separately showed a significant decrease in IL-6 and TNFα gene expression compared to controls. However, the fixed combination of Citicoline, CoQ10 (CAVAQ10), and vitamin B3 had greater synergistic effects, leading to the proposal that a stronger therapeutic approach should be adopted to lessen neurodegeneration and inflammation in neuronal conditions like glaucoma [85].

The anti-inflammatory properties of Citicoline can be attributed to several mechanisms. These include the inhibition of NF-κB activation, increased activity of PPAR-γ, elevated levels of acetylcholine, inhibition of MAPK activation (such as p38 MAPK and JNK), reduction in other MAPKs like ERK, and regulation of the JAK/STAT signaling pathway by inhibiting JAK2 and STAT3 activation. These pathways regulate pro-inflammatory gene expression, leading to decreased pro-inflammatory cytokine production [84,86,87,88,89,90,91]. Similarly, the anti-inflammatory properties of Coenzyme Q10 can be attributed to the activation of the neuroglial NLRP3 inflammasome [92], and the inhibition of MAPK and NF-κB signaling pathways, which can reduce neuroinflammation and modify microglial polarization pathways [93,94]. Based on that evidence, this drug combination may provide neuroprotective properties through the inhibition of the pathological progression in neuroinflammation-related diseases.

## 4. Materials and Methods

### 4.1. Animals

In this study, 60 CD-1 Swiss albino male mice, aged 12–16 weeks and weighing 35–45 g, were utilized. The animals were sourced from Charles River Laboratory (Barcelona, Spain) and housed in the animal facility of the School of Medicine at Complutense University of Madrid (Spain). They were maintained under controlled light (12 h light/dark cycles, 9–24 lux) and temperature conditions, with free access to a standard diet and water. Experiments were conducted in accordance with ethical guidelines endorsed by Spanish legislation and the Guidelines for Humane Endpoints for Animals Used in Biomedical Research. This study was approved by the Animal Research Ethics Committee of Complutense University (Madrid, Spain) and the General Directorate of Agriculture and Food of the Ministry of Economy and Employment of the Community of Madrid (PROEX 091.2/22). All experimental procedures adhered to institutional guidelines, European Union regulations for the use of animals in research, and the ARVO (Association for Research in Vision and Ophthalmology) Statement for the Use of Animals in Ophthalmic and Vision Research. Additionally, this work was written up following the ARRIVE guidelines (Animal Research: Reporting of In Vivo Experiments), which provided recommendations for describing and publishing studies carried out using animals for experimental purposes [95].

### 4.2. Experimental Groups

**Figure 24:**
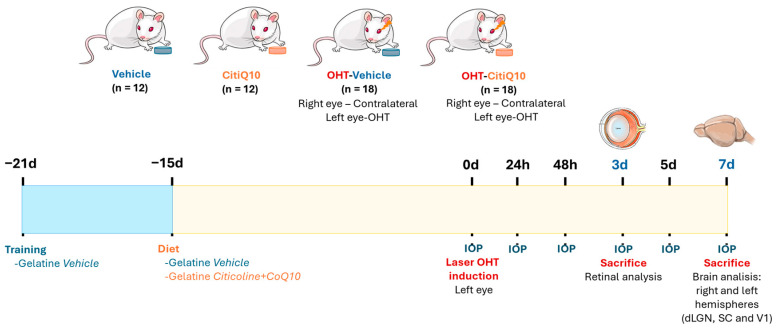
Scheme of the experimental design and groups of this study. Abbreviations: CitiQ10: Citicoline and CoQ10; OHT: ocular hypertension; IOP: intraocular pressure; dLGN: dorsolateral geniculate nucleus; SC: superior colliculus; V1: primary visual cortex; h: hours; d: days.

The animals were categorized into four experimental groups: (1) vehicle group (n = 12; 6 for retinas and 6 for brains), which consisted of mice that were given neutral gelatin for the duration of the study period without any procedures performed; (2) Citicoline + CoQ10 (CitiQ10) group (n = 12; 6 for retinas and 6 for brains), where mice were given Citicoline and CoQ10 throughout the study period without undergoing any procedures; (3) laser OHT-vehicle group (OHT) (n = 18; 12 for retinas and 6 for brains), where mice were administered neutral gelatin during the study and underwent induced OHT; and (4) laser OHT-Citicoline+CoQ10 group (OHT-CitiQ10) (n = 18; 12 for retinas and 6 for brains), in which mice were given Citicoline and CoQ10 during the study period and were exposed to induced OHT. In both OHT and OHT-CitiQ10 groups, hypertensive eyes (left eye, OHT) and their contralateral counterparts (right eye, contralateral) were examined 3 days after laser-induced OHT. For the rest of the visual pathway study, the dLGN, the SC, and V1 from both the right (dLGN right, SC right, V1 right) and left (dLGN left, SC left, V1 left) hemispheres were analyzed in all experimental groups 7 days after OHT induction (Figure 24).

### 4.3. Citicoline and CoQ10 Treatment

The preparation and administration of Citicoline + CoQ10 was carried out according to a previous protocol [7]. The oral administration of Citicoline and CoQ10 was provided daily through a 0.5 mL gelatin capsule, which was formulated in our laboratory utilizing commercially available porcine skin gelatin (Sigma-Aldrich G2500-500G, Merck KGaA, Darmstadt, Alemania) and potable water. For the treatment group, the gelatin contained Citicoline (Neurotidine^®^, Omikron Pharmaceutical España S.L.U., Barcelona, Spain) at a dose of 500 mg/kg and CoQ10 (COQUN^®^ OS, VISUfarma B.V., Madrid, Spain) at a dose of 200 mg/kg. This combination (Citicoline + CoQ10) is trade marketed as COQUN Combo (COQUN^®^ Combo, VISUfarma S.p.A., Rome, Italy). The daily dose was used by Matamoros et al., 2025 [7], taking as reference an average animal weight of 40 g. Control group animals received only gelatin as a vehicle. The administration of gelatin (with or without the compound) commenced 15 days before OHT induction and persisted until the designated sacrifice points (3 or 7 days post-OHT induction), following the protocol by Fernández-Albarral et al., 2021 [36]. The person administering the gelatin ensured the animals ingested it completely by monitoring them until it was fully consumed.

To ensure full consumption during the experimental phase, all experimental groups were trained for 1 week with a daily gelatin dose of 0.5 mL (made from potable water and commercial gelatin, gelatin from porcine skin, Sigma-Aldrich G2500-500G) before starting gelatin administration with either the compound or the vehicle, to familiarize the animals with the ingestion process (Figure 24).

### 4.4. Anesthesia

The anesthesia procedure was carried out according to previous protocols [7]. General anesthesia was administered for the surgical procedures, including OHT induction and animal euthanasia, through intraperitoneal injections of ketamine (Anestekin^®^ 100 mg/mL, Dechra Veterinary Products SLU, Barcelona, Spain), medetomidine (Dormisan^®^ 1 mg/mL, Fatro Ibérica, Barcelona, Spain), and saline solution. To promote recovery from anesthesia, 0.1 mL of atipamezole hydrochloride (Nosedorm^®^ 5 mg/mL, Laboratorios Karizoo S.A., Barcelona, Spain) was administered subcutaneously. The cornea was treated with a local anesthetic (COLICURSÍ™ ANESTÉSICO DOBLE, 1 mg/mL tetracaine hydrochloride and 4 mg/mL oxybuprocaine hydrochloride, Alcon España, Barcelona, Spain) for OHT induction. IOP measurements were carried out under inhalational anesthesia with 2% isoflurane in oxygen (Isoflutek^®^ 1000 mg/g, Laboratorios Karizoo S.A., Barcelona, Spain).

### 4.5. Induction of Ocular Hypertension and IOP Measurement

We followed the protocol established by Ramirez et al., 2023 [33] (Viridis Ophthalmic Photocoagulator—532 nm, Quantel Medical, Clermont-Ferrand, France) targeting the limbal and episcleral veins. The parameters used were a spot size of 50 µm, a power setting of 0.3 W, and a duration of 0.5 s. The mice received an average of 80–150 laser impacts, administered without any lens. To prevent corneal dryness, inflammation, and infection, a drop of Tobradex^®^ (1 mg/mL dexamethasone and 3 mg/mL tobramycin, Alcon, Barcelona, Spain) was applied after OHT induction. Following the anesthesia protocol mentioned above, IOP was measured as previously described [36,39], using a rebound tonometer (TonoLab, Tiolat, OY, Helsinki, Finland). IOP was evaluated in both the OHT and contralateral eyes of mice from all experimental groups. Each IOP measurement was the average of three independent readings, with each reading being the automatic mean of six consecutive measurements. Baseline IOP was noted at the start of the experiment. Subsequent to the induction of OHT, IOP was measured at five additional time points: 24 h, 48 h, 3 days, 5 days, and 7 days post-OHT induction. All IOP measurements were conducted at the same time each day (10:00 AM) to minimize variability attributable to circadian rhythms (Figure 24).

### 4.6. Immunohistochemistry

The tissue processing was carried out following the protocols of previous studies [7]. Following an overdose of the general anesthesia described in the anesthesia section, the animals were sacrificed. Transcardiac perfusion was performed on the mice, beginning with a saline solution (0.9% NaCl) with heparin (0.2 mL/L heparin 5000 UI/mL, Reig Jofré, S.A.) followed by a 4% paraformaldehyde (PFA) solution in 0.1 M phosphate buffer. The solutions were administered at a volume of 1500 mL/g of body weight and kept at 4 °C. Following perfusion, a suture was placed in the upper eyelid to secure the retina’s spatial orientation. The eyes and brains were harvested and placed in 4% PFA at 4 °C for 24 h. For the eyes, the corneas and lenses were removed, and the retinas were isolated to create retinal whole-mounts. The retinas underwent cryoprotection by immersion in sucrose solutions with progressively increasing concentrations (10%, 20%, and 30%) for 1 h, 2 h, and overnight, respectively, at 4 °C. Afterwards, the tissues were frozen using liquid nitrogen and stored at −80 °C until further use. Post-fixation, the brains were rinsed three times in 0.1 M PBS and then cryoprotected by rinsing for 48 h at 4 °C in 11% sucrose in PBS 0.1 M, followed by 33% sucrose in PBS 0.1 M. The brains were then included in Tissue Freezing Medium (Tissue-Tek O.C.T., Sakura Finetek Japan Co., Ltd., Tama, Tokio, Japan)) and stored at −20 °C until used [16].

#### 4.6.1. Immunohistochemistry in Retinal Tissue

Several primary antibodies were utilized for immunohistochemical examination. For the analysis of morphological signs of microglial activation, we used a widely employed antibody for labeling microglial cells, Iba-1 (ionized calcium-binding adaptor molecule 1) [96]. We also employed the antibody against the P2 purinergic receptor P2RY12, which allows for the differentiation of microglia from other cells such as macrophages or infiltrating monocytes. Additionally, this antibody is capable of distinguishing between resting and activated microglia [97]. To label macroglial changes (astrocytes and Müller glia), we used an antibody targeting glial fibrillary acidic protein (GFAP), as this protein constitutes the intermediate filaments of the astrocyte cytoskeleton [22]. The retinas were immunostained according to previously used protocols [23]. Briefly, retinas were double immunostained with rabbit anti-Iba-1 (1:600; Wako, Neuss, Germany, 019-19741) and rat anti-P2RY12 (1:100; Biolegend, San Diego, CA, USA, 848002) followed by a secondary antibody, donkey anti-rabbit IgG Alexa Fluor 594 (1:800; Invitrogen, Atlanta, GA, USA, A21207), and goat anti-rat IgG Alexa Fluor 488 (1:150; Biolegend, 405418). In addition, retinas also were immunostained with mouse anti-GFAP clone GA5 (1:150; Millipore, Frankfurt am Main, Germany, MAB3402) followed by a secondary antibody, goat anti-mouse IgG Alexa Fluor 594 (1/250; Invitrogen, A11020). To test the specificity of the antibodies used, we performed three negative controls. In the first control, no primary antibody was added. In the second control, the secondary antibody was omitted. Finally, in the third control, neither primary nor secondary antibodies were added, so the retinas were incubated only with the corresponding diluents, allowing us to observe the levels of endogenous fluorescence. To study and photograph the retinas, we utilized an ApoTome 2 system (Carl Zeiss, Oberkochen, Germany) along with an Axio CAM 503 Mono high-resolution digital camera (Carl Zeiss), both integrated with a Zeiss Axio Imager M.2 fluorescence microscope (Carl Zeiss), as previously described [18]. The microscope was fitted with suitable filters for various emission spectra, specifically Alexa Fluor 594 (Filter set 64, Zeiss) and Alexa Fluor 488 (Filter set 10, Zeiss). The ApoTome device enables the capture of high-quality images of thick tissues, which can be challenging due to fluorescence signals outside the focal plane, leading to reduced contrast and resolution along the axial dimension (z-axis). The ApoTome enhances imaging by simulating optical sectioning, thereby improving image contrast and resolution. It projects a grating in the focal plane of the objective, which moves to three different positions on the sample, utilizing the principles of interferometry. The microscope’s ZEN2 software (Carl Zeiss AG, Oberkochen, Germany) processes the images by eliminating out-of-focus elements, resulting in higher-quality images that resemble optical sections in the plane of focus.

#### 4.6.2. Immunohistochemistry in Visual Pathway Nuclei

Brains were sectioned into 20 μm thick coronal slices using a cryostat (Leica, Nussloch, Germany, CM3050). We focused on three specific regions of the visual pathway, namely, dLGN, located between Bregma −1.855 mm and −2.88 mm; the SC, Bregma −3.08 mm to −3.98 mm; and the V1, Bregma −2.78 mm to −3.78 mm, according to the Allen Brain Atlas (available online at mouse.brain-map.org). Three sections per slide were mounted on gelatin-coated slides, air-dried for a minimum of 30 min, and subsequently stored at −30 °C. Brain sections were immunostained according to previous protocols from our research group [16]. Briefly, an immunohistochemistry buffer (IB, 0.5% Animal-Free Blocker^®^ and Diluent, R.T.U., Vector Laboratories, Inc., Newark, CA, USA, Ref. SP-5035, and 0.3% Triton X-100, Sigma-Aldrich, St. Louis, MO, USA, Ref. T8787 in 0.1 M PBS, Sigma-Aldrich, USA, Ref. P4417), at pH 7.4, was used for washes and incubations. IB containing 0.5% H_2_O_2_ was used for 15 min at room temperature (RT) to block the endogenous peroxidase. Brain sections were washed with IB three times and then incubated in IB, overnight at 4 °C, with specific markers for microglial and macroglial cells, Iba1 (1:500; Wako, 1919471), and GFAP (1:800; Dako, Carpinteria, CA, USA, Z0334), respectively. Thereafter, slides were rinsed in IB, three times, and then incubated with the secondary antibody for 2 h at RT (Goat-Anti-rabbit IgG (H + L) Biotinylated, 1:200; Thermo Scientific, Atlanta, GA, USA, 31820). Sections were then washed in IB, three times, and incubated with the avidin-biotin peroxidase complex (Vectastain ABC kit, 1:250; Thermo Scientific, #32020) for 90 min at RT. Brain sections from a total of 5 animals per experimental group and sacrifice day were randomly chosen for the immunohistochemical analyses and, in each immunohistochemical assay, slides from all the experimental groups and brain areas of interest were included. A negative control slide with no primary antibody was also considered. Immunostained slides were observed under a light microscope (Zeiss Axioplan Microscope, Oberkochen, Germany) equipped with a high-resolution camera (Zeiss Axioplan 712 color, Germany), and microphotographs were taken and processed using the ZEN3.3 software, version 3.3.89.0000 (Carl Zeiss AG, Oberkochen, Germany). To ensure uniformity in the photography process, the shine, contrast, light, and magnification were kept constant during image capture. Microphotographs were processed for presentation by using Adobe Photoshop CS4 Extended 10.0 (Adobe Systems, San Jose, CA, USA).

### 4.7. Quantitative Analysis

#### 4.7.1. Retina

##### Iba-1+ Cells

To assess the impact of OHT on retinal Iba-1+ cells and the potential anti-inflammatory effects of the combination of Citicoline and CoQ10, we measured several parameters 3 days after laser-induced OHT: (i) the number of Iba-1+ cells in the outer segment (OS), outer plexiform layer (OPL), and inner plexiform layer (IPL); (ii) the area of the retina occupied by Iba-1+ cells (Iba1-RA) in the nerve fiber layer–ganglion cell layer (NFL-GCL); (iii) the arbor area of Iba-1+ cells in the OPL and IPL; (iv) the number of microglial vertical processes connecting the OPL and OS; and (v) the cell body area of Iba-1+ cells in the OPL, IPL, and NFL-GCL. All measurements were conducted in a double-blind manner. The OS, OPL, IPL, and NFL-GCL were distinguished based on microglia morphological differences.

Quantification was carried out across all experimental groups, following our group’s previous methodology [23]. To analyze the number of Iba-1+ cells, equivalent retinal areas were consistently selected and photographed for each retinal whole-mount in both vertical and horizontal meridians intersecting the optic nerve (covering superior, inferior, nasal, and temporal zones). All analyzed fields were contiguous to ensure no part of the retinal whole-mount was omitted or duplicated. Each meridian was scanned along its entire length using the microscope’s motorized stage along the x-y axis, resulting in approximately 550 fields evaluated [23]. These areas were photographed at 20× magnification, covering an area of 0.1502 mm^2^ per field. Additionally, to quantify Iba-1+ cells in the OPL, IPL, and NFL-GCL, the entire preparation was analyzed along the z-axis in depth, with sections taken every 2 μm.

The quantification method varied based on the cell number and distribution characteristics in each retinal layer. In the IPL and OPL, Iba-1+ cells formed a cellular network and were distributed in a non-overlapping mosaic pattern, enabling individual identification and automatic cell counting [31]. However, in the NFL-GCL and OS, the distribution of Iba-1+ cells was irregular, not allowing automatic counting. Therefore, in the NFL-GCL, we quantified the Iba1-RA [98,99], and in the OS, we performed manual quantification.

Number of Iba-1+ cells in the OS

To quantify Iba-1+ cells in this layer, we utilized the “Interactive” manual counting tool in the ZEN2 software (Carl Zeiss), along with the ApoTome device attached to the fluorescence microscope.

Number of Iba-1+ cells in the OPL, IPL, and NFL-GCL

To count the Iba-1+ cells in the OPL and IPL, as well as Iba1-RA cells in the NFL-GCL, we used established protocols with an automatic segmentation algorithm and distance control developed by us in the MATLAB environment (R2023b, MathWorks, Natick, MS, USA) [99]. In summary, the counting protocol was as follows: the images to be quantified were averaged in a Z-stack, creating a Z projection, which was processed in two ways to preserve only the most intense parts of the Iba-1+ cells (the soma). This feature was used for the identification and subsequent quantification of the Iba-1+ cells [98,99]. First, the image was normalized to the pixel with the highest value, so that the image values ranged between 0 and 1. Subsequently, a threshold was applied, setting all values below 0.2 to 0, while retaining the remaining values. The resulting image was segmented, and the center of mass of each segment was determined to identify the presence or absence of cells. To avoid counting the same cell twice in adjacent segments, a minimum separation distance was specified. Points separated by a distance less than the established threshold were considered part of the same cell and were counted only once.

In the NFL-GCL, we assessed Iba1-RA levels in each chosen image using a MATLAB threshold tool. This tool set the grayscale value range for pixels containing the target objects, separating them from other image regions. After adjusting the threshold, we applied the “count NFL” algorithm to determine the percentage of retinal area marked with anti-Iba-1.

Arbor area of Iba-1+ cells in the OPL and IPL

For the quantification of the arbor area, we followed the protocol employed in previous studies [23]. From the retinal areas used for counting Iba-1+ cells in the plexiform layers of the retinal whole-mounts, four areas were selected in each layer. These areas represented different regions of the retina and were taken at specific distances from the optic disc in various retinal quadrants: in the superior retina, the area closest to the optic disc; in the inferior retina, the area located at two levels of eccentricity from the optic disc; in the nasal retina, the area located at three levels of eccentricity; and in the temporal retina, the area situated at four levels of eccentricity. In the selected 20× photomicrographs, a polygon was manually drawn by connecting the distal-most tips of the Iba-1+ cell processes using the “Interactive Measurement” tool in ZEN2 software (Carl Zeiss) along with the ApoTome device attached to the fluorescence microscope. Subsequently, a computer-assisted morphometric algorithm quantified the arbor area of Iba-1+ cells [23].

Number of vertical processes of Iba-1+ cells connecting the OPL and OS

In each of the four selected retinal regions used for arbor area quantification, we obtained 20× magnification photomicrographs of the vertical processes of Iba-1+ cells situated between the outer plexiform layer (OPL) and the outer segments (OS), which are observed as dots in the image. The quantification of these dots was performed manually using the ZEN2 software.

Cell body area of Iba-1+ cells in the OPL, IPL, and NFL-GCL

In the areas where the arbor area and vertical processes were quantified, we captured 20× magnification images in the OPL, IPL, and NFL-GCL. We traced the contours of the Iba-1+ cell bodies using the “Interactive Measurement Tool” in ZEN2 software.

##### Microglial P2RY12 Expression

To evaluate the effect of OHT on microglial P2RY12 expression and the potential anti-inflammatory role of the combined treatment with citicoline and CoQ10, the P2RY12-labeled retinal area (P2RY12-RA) was determined 3 days after laser-induced OHT, similar to Iba1-RA quantification.

In the NFL-GCL, equivalent retinal areas were methodically chosen and photographed for each retinal whole-mount, following the same method as for obtaining the images for the Iba1+ cells analysis. We measured P2RY12-RA in each chosen image using the MATLAB threshold tool, calculating the percentage of retinal area marked by anti-P2RY12.

##### GFAP+ Cells

To assess the impact of OHT on retinal GFAP+ cells (astrocytes and activated Müller glia) and the potential anti-inflammatory effects of the combination of Citicoline and CoQ10, the GFAP-labeled retinal area (GFAP-RA) was quantified 3 days after laser-induced OHT.

Photographs were captured across all experimental groups to quantify GFAP-RA in retinal whole-mounts, following our group’s previous methodology [22]. In the retinal whole-mounts, images were captured along the entire X-Y axis using the automated movement of the motorized stage, ensuring contiguous fields without duplication or omission of parts of the whole-mount. The photomicrographs were taken at 20× magnification, providing an area of 0.1502 mm^2^ per field. Approximately 550 fields were analyzed. For the GFAP-RA quantification, a threshold tool in MATLAB was applied to microphotographs. The pixels corresponding to the objects of interest were identified using thresholds based on grayscale values, allowing them to be distinguished from other areas of the image. Subsequently, GFAP-RA was quantified using an algorithm developed by our group in the MATLAB environment [99].

#### 4.7.2. Rest of the Visual Pathway

##### Iba-1+ Cells

Iba-1+ cell analysis was performed on microphotographs taken of sections observed under light microscopy at 10× magnification, by two blinded observers (inter-observer differences <15%). Light, brightness, and contrast conditions were kept constant during the capture process. The three nuclei of interest were analyzed in both hemispheres (dLGN right, dLGN left, SC right, SC left, V1 right and V1 left). Using the ImageJ software (version 1.46r Research Services Branch-NIH, Bethesda, MD, USA), the number of Iba-1+ cells was counted as well as the number of cells of each morphotype, distinguishing between resting morphotypes (types I and II) and activated morphotypes (types III, IV and V) in accordance with Gomes et al., 2015 [100]. In each microphotograph, a grid was established in the area of interest, and six squares (randomly selected) of approximately 0.07 mm^2^ were counted; therefore, a total surface of 0.42 mm^2^ was studied in each area. Results were expressed as the number of Iba1+ cells per unit of area in each brain region (n°/mm^2^), and as the percentage of active morphotypes in relation to the total number of Iba-1+ cells in each brain region (%).

##### GFAP+ Cells

GFAP+ expression quantification was performed on microphotographs, captured at 10× magnification, by using digital optical densitometry (ImageJ software, version 1.46r (Research Services Branch-NIH, Bethesda, MD, USA). The three nuclei of interest were analyzed in both hemispheres (dLGN right, dLGN left, SC right, SC left, V1 right and V1 left), and all the quantifications were performed by two blinded observers (inter-observer differences <15%). The regions of interest (ROIs) were situated randomly in the areas of interest for densitometric study: in dLGN, we analyzed a total surface in the central zone of 0.0347 mm^2^ and, in the peripheral, one of 0.0222 mm^2^; in SC, a total area of 0.0417 mm^2^; and in V1, a total area of 0.111 mm^2^. Densitometric values, expressed as optical density (O.D.) in arbitrary units, referred to the surface studied.

### 4.8. Statistical Analysis

Statistical analysis was conducted using GraphPad Prism 9.0 (GraphPad Prism, La Jolla, CA, USA). For the retinal study, statistical significance among the groups (vehicle, CitiQ10, OHT, contralateral, OHT-CitiQ10, and contralateral-CitiQ10) was evaluated using the Mann–Whitney U test for unpaired data or the Wilcoxon signed-rank test for comparisons between OHT and contralateral eyes. The parameters compared included (i) the IOP; (ii) the Iba-1 + cell number in the OS, OPL, and IPL; (iii) Iba1-RA in the NFL-GCL; (iv) the arbor area of Iba-1 + cells in the OPL and IPL; (v) the number of microglial vertical processes connecting the OPL and OS; (vi) the cell body area of Iba-1 + cells in the OPL, IPL, and NFL-GCL; (vii) the P2RY12-RA in the NFL-GCL, and (viii) the GFAP-RA in the NFL-GCL. A *p*-value of <0.05 was considered statistically significant.

For the visual pathway study, statistical significance among the groups (vehicle, CitiQ10, laser-OHT, and laser-OHT-CitiQ10) in the dLGN, the SC, and V1 from both the right (OHT dLGN right, OHT SC right, OHT V1 right) and left (contralateral dLGN left, contralateral SC left, contralateral V1 left) hemispheres were assessed using the Mann–Whitney U test for unpaired data or the Wilcoxon signed-rank test for comparisons between left and right hemispheres. The parameters compared were the (i) Iba-1+ cell number and (ii) GFAP+ expression. A *p*-value of <0.05 was considered statistically significant.

The effect size was calculated using Pearson’s r, with an absolute value of r ≈ 0.5 considered indicative of a robust effect size (Appendix A).

## 5. Conclusions

In conclusion, the present study demonstrates that the combination of Citicoline and Coenzyme Q10 can produce anti-inflammatory effects against the damage caused by increased IOP in retinal tissue and along the visual pathway. Therefore, this combination may help protect RGCs from the inflammatory process generated in glaucomatous pathology.

## Data Availability

The data presented in this study are available on request from the corresponding author. The data are not publicly available due to patentability concerns.

## Abbreviations

BAX	BCL-2-associated X protein
BCL-2	B-cell lymphoma 2
BDNF	Brain-derived neurotrophic factor
BRB	Blood retinal barrier
CD200R	CD200 receptor
dLGN	Dorsolateral geniculate nucleus
CNS	Central nervous system
CoQ10	Coenzyme Q10
CRLS1	Cardiolipin synthase 1
CXCR1	CX3C chemokine receptor 1
ERK	Extracellular signal regulated kinase
GFAP	Glial fibrillary acidic protein
GFAP-RA	Retinal area occupied by GFAP+ expression
GCL	Ganglion cell layer
H_2_O_2_	Hydrogen peroxide
Iba 1-RA	Retinal area occupied by Iba-1+ expression
IL-1β	Interleukin 1 beta
IL-6	Interleukin 6
IL-8	Interleukin 8
IFN- γ	Interferon gamma
iNOS	Inducible nitric oxide synthase
IOP	Intraocular pressure
IPL	Inner plexiform layer
JAK2	Janus kinase 2
JNK	c-Jun N-terminal kinase
LGN	Lateral geniculate nucleus
MAPK	Mitogen-activated protein kinase
NF-κB	Nuclear factor kappa B
NFL	Never fiber layer
NLRP3	NOD-like receptor family pyrin domain containing 3
OHT	Ocular hypertension
OPL	Outer plexiform layer
OS	Outer segment
pERK	Phosphorylated ERK
PPAR- γ	Peroxisome proliferator-activated receptor gamma
P2RY12	Purinergic receptor P2RY12
P2RY12-RA	Retinal area occupied by P2RY12+ expression
RGCs	Retinal ganglion cells
ROS	Reactive oxygen species
SC	Superior colliculus
SD	Standard deviation
STAT3	Signal transducer and activator of transcription 3
SOD2	Superoxide dismutase 2
TNF-α	Tumor necrosis factor alpha
VEGF	Vascular endothelial growth factor
VP	Vertical processes
V1	Primary visual cortex

## Appendix A. Estimation of the Size Effect in Statistically Significant Comparisons

**Table A1:** Intraocular pressure.

IOP						
		Vehicle vs. OHT	OHT vs. Contralateral	CitiQ10 vs. OHT-CitiQ10	OHT-CitiQ10 vs. Contralateral-CitiQ10	OHT vs. OHT-CitiQ10
**24 h**	Effect size |r|	0.74	0.57	0.23	0.56	0.34
	CI (95%)	(15.00, 17.00)	(−17.00, −13.00)	(10.00, 12.00)	(−13.00, −9.00)	(−6.00, −2.00)
	*p*-value	<0.0001	<0.0001	<0.0001	<0.0001	0.0006
**48 h**	Effect size |r|	0.74	0.56	0.74	0.58	
	CI (95%)	(12.00, 18.00)	(−19.00, −10.00)	(10.00, 13.00)	(−16.00, −10.00)	
	*p*-value	<0.0001	<0.0001	<0.0001	<0.0001	
**3d**	Effect size |r|	0.92	0.56	0.9	0.56	0.31
	CI (95%)	(11.00, 16.00)	(−18.00, −8.00)	(5.00, 8.00)	(−11.00, −6.00)	(−8.00, −1.00)
	*p*-value	<0.0001	<0.0001	<0.0001	<0.0001	0.0089
**5d**	Effect size |r|	0.95	0.95	0.73	0.96	
	CI (95%)	(5.00, 8.00)	(−11.00, −5.00)	(4.00, 16.00)	(−15.00, −3.00)	
	*p*-value	<0.0001	0.0005	<0.0001	0.0001	
**7d**	Effect size |r|	0.3	0.95	0.23	0.96	
	CI (95%)	(2.00, 5.00)	(−15.00, −4.00)	(1.00, 3.00)	(−5.00, −2.00)	
	*p*-value	<0.0001	0.0005	0.0024	0.0002	

OHT: ocular hypertension; r: Pearson’s r; CI: 95% confidence interval; h: hours; d: days.

**Table A2:** Iba-1+ cells.

	Iba-1+ Cells								
		Vehicle vs. OHT	Vehicle vs. Contralateral	OHT vs. Contralateral	CitiQ10 vs. OHT-CitiQ10	CitiQ10 vs. Contralateral-CitiQ10	OHT-CitiQ10 vs. Contralateral-CitiQ10	OHT vs. OHT-CitiQ10	Contralateral vs. Contralateral-CitiQ10
**OS**	Effect size |r|	0.73		0.33	0.4		0.33	0.39	0.14
	CI (95%)	(7.00, 10.00)		(−11.00, −6.00)	(2.00, 4.00)		(−4.00, −1.00)	(−7.00, −4.00)	(−1.00, 0.00)
	*p*-value	<0.0001		<0.0001	<0.0001		<0.0001	<0.0001	0.0177
**OPL**	Effect size |r|	0.72		0.34	0.79	0.35	0.35	0.14	
	CI (95%)	(17.00, 21.00)		(−26.00, −22.00)	(16.00, 19.00)	(−4.00, −2.00)	(−23.00, −19.00)	(−5.00, 0.00)	
	*p*-value	<0.0001		<0.0001	<0.0001	<0.0001	<0.0001	0.0279	
**IPL**	Effect size |r|	0.86	0.22	0.34	0.84	0.14	0.33	0.34	
	CI (95%)	(34.00, 37.00)	(−3.00, −1.00)	(−40.00, −35.00)	(25.00, 29.00)	(−2.00, 0.00)	(−30.00, −26.00)	(−12.00, −7.00)	
	*p*-value	<0.0001	0.0008	<0.0001	<0.0001	0.0201	<0.0001	<0.0001	

OHT: ocular hypertension; OS: outer segment; OPL: outer plexiform layer; INL: Inner plexiform layer; r: Pearson’s r; CI: 95% confidence interval.

**Table A3:** Iba-1+ RA.

Iba-1+ RA- NFL-GCL									
	Vehicle vs. CitiQ10	Vehicle vs. OHT	Vehicle vs. Contralateral	OHT vs. Contralateral	CitiQ10 vs. OHT-CitiQ10	CitiQ10 vs. Contralateral-CitiQ10	OHT-CitiQ10 vs. Contralateral-CitiQ10	OHT vs. OHT-CitiQ10	Contralateral vs. Contralateral-CitiQ10
Effect size |r|	0.18	0.86	0.18	0.34	0.86	0.19	0.36	0.75	0.22
CI (95%)	(−1324, −230.3)	(30,482, 34,480)	(310.4, 1618)	(−34,390, −29,284)	(16,279, 18,092)	(255.8, 1309)	(−18,272, −15,566)	(−18,798, −14,635)	(−1549, −441.0)
*p*-value	0.0048	<0.0001	0.0034	<0.0001	<0.0001	0.0034	<0.0001	<0.0001	0.0006

OHT: ocular hypertension; RA: area of the retina; NFL-GCL: nerve fiber layer–ganglion cell layer; r: Pearson’s r; CI: 95% confidence interval.

**Table A4:** Iba-1+ arbor area.

Iba-1+ Arbor Area							
		Vehicle vs. OHT	OHT vs. Contralateral	CitiQ10 vs. OHT-CitiQ10	CitiQ10 vs. Contralateral-CitiQ10	OHT-CitiQ10 vs. Contralateral-CitiQ10	OHT vs. OHT-CitiQ10
**OPL**	Effect size |r|	0.81	0.26	0.76	0.1	0.26	0.47
	CI (95%)	(−3350, −2989)	(−2922, 3703)	(−2770, −2441)	(−523.6, −12.93)	(2039, 2544)	(587,4, 771,1)
	*p*-value	<0.0001	<0.0001	<0.0001	0.0395	<0.0001	<0.0001
**IPL**	Effect size |r|	0.7	0.33	0.71		0.31	0.51
	CI (95%)	(−4853, −4216)	(4005, 4628)	(−3808, −3295)		(−3066, 3782)	(514.0, 664.4)
	*p*-value	<0.0001	<0.0001	<0.0001		<0.0001	<0.0001

OHT: ocular hypertension; OPL: outer plexiform layer; INL: inner plexiform layer; r: Pearson’s r; CI: 95% confidence interval.

**Table A5:** Iba-1+ cell body area.

Iba-1+ Cell Body Area										
		Vehicle vs. CitiQ10	Vehicle vs. OHT	Vehicle vs. Contralateral	OHT vs. Contralateral	CitiQ10 vs. OHT-CitiQ10	CitiQ10 vs. Contralateral-CitiQ10	OHT-CitiQ10 vs. Contralateral-CitiQ10	OHT vs. OHT-CitiQ10	Contralateral vs. Contralateral-CitiQ10
**OPL**	Effect size |r|	0.44	0.85		0.46	0.77		0.46	0.79	0.37
	CI (95%)	(−20.35, −9.997)	(72.45, 98.21)		(−99.40, −74.46)	(35.14, 45.45)		(−44.06, −33.44)	(−73.27, −48.23)	(−15.15, −6.235)
	*p*-value	<0.0001	<0.0001		<0.0001	<0.0001		<0.0001	<0.0001	<0.0001
**IPL**	Effect size |r|	0.5	0.85	0.3	0.46	0.73	0.29	0.46	0.83	
	CI (95%)	(−22.83, −11.80)	(86.10, 108.40)	(−1.085, −0.5668)	(−116.9, −94.04)	(27.98, 38.88)	(3.659, 11.85)	(−34.27, −21.33)	(−92.19, −71.06)	
	*p*-value	<0.0001	<0.0001	0.0313	<0.0001	<0.0001	0.0004	<0.0001	<0.0001	
**GCL-NFL**	Effect size |r|	0.22	0.86	0.2	0.46	0.75	0.43	0.47	0.8	
	CI (95%)	(−11.59, −1.340)	(140.9, 179.3)	(1.082, 11.59)	(−180.0, −130.7)	(37.15, 52.71)	(9.224, 18.50)	(−41.12, −23.45)	(−143.5, −105.8)	
	*p*-value	0.0114	<0.0001	0.0208	<0.0001	<0.0001	<0.0001	<0.0001	<0.0001	

OHT: ocular hypertension; OPL: outer plexiform layer; INL: inner plexiform layer; NFL-GCL: nerve fiber layer–ganglion cell layer; r: Pearson’s r; CI: 95% confidence interval.

**Table A6:** Vertical processes.

VP							
	Vehicle vs. OHT	Vehicle vs. Contralateral	OHT vs. Contralateral	CitiQ10 vs. OHT-CitiQ10	CitiQ10 vs. Contralateral-CitiQ10	OHT vs. OHT-CitiQ10	Contralateral vs. Contralateral-CitiQ10
Effect size |r|	0.35	0.84	0.79	0.71	0.32	0.42	0.71
CI (95%)	(0.00, 4.00)	(14.00, 32.00)	(9.00, 33.00)	(4.00, 7.00)	(0.00, 6.00)	(1.00, 5.00)	(−28.00, −13.00)
*p*-value	0.0186	<0.0001	<0.0001	<0.0001	0.0271	0.0027	<0.0001

OHT: ocular hypertension; VP: vertical processes; r: Pearson’s r; CI: 95% confidence interval.

**Table A7:** P2RY12-RA.

P2RY12-RA				
	Vehicle vs. OHT	OHT vs. Contralateral	CitiQ10 vs. Contralateral-CitiQ10	OHT vs. OHT-CitiQ10
Effect size |r|	0.84	0.84	0.32	0.07
CI (95%)	(−99.47, −68.19)	(−4949, 8372)	(−3054, −133.7)	(−3514, 7536)
*p*-value	<0.0001	<0.0001	0.0287	<0.0001

OHT: ocular hypertension; P2RY12-RA; retinal area occupied by P2RY12+ expression; r: Pearson’s r; CI: 95% confidence interval.

**Table A8:** GFAP-RA.

GFAP-RA									
	Vehicle vs. CitiQ10	Vehicle vs. OHT	Vehicle vs. Contralateral	OHT vs. Contralateral	CitiQ10 vs. OHT-CitiQ10	CitiQ10 vs. Contralateral-CitiQ10	OHT-CitiQ10 vs. Contralateral-CitiQ10	OHT vs. OHT-CitiQ10	Contralateral vs. Contralateral-CitiQ10
Effect size |r|	0.25	0.86	0.64	0.33	0.76	0.41	0.36	0.76	0.8
CI (95%)	(2302, 6988)	(66.101, 72.030)	(14.569, 20.288)	(−52.951,−45.181)	(19.824, 26,605)	(5701, 9918)	(−20.014, −8946)	(−44.106, −36.074)	(−7687,−2113)
*p*-value	0.0001	<0.0001	<0.0001	<0.0001	<0.0001	<0.0001	<0.0001	<0.0001	0.0005

OHT: ocular hypertension; GFAP-RA; retinal area occupied by GFAP+ expression; r: Pearson’s r; CI: 95% confidence interval.

**Table A9:** Iba-1 dLGN.

Iba-1 dLGN						
		Vehicle vs. OHT dLGN Right	Vehicle vs. Contralateral dLGN Left	CitiQ10 vs. OHT-CitiQ10 dLGN Right	OHT dLGN Right vs. OHT-CitiQ10 dLGN Right	Contralateral dLGN Left vs. Contralateral-CitiQ10 dLGN Left
**Total**	Effect size |r|	0.03				
	CI (95%)	(−6.12, −3.88)				
	*p*-value	0.0319				
**Actived morphotypes**	Effect size |r|	0.81	0.81	0.77	0.83	0.83
	CI (95%)	(16.38, 18.62)	(7.44, 9.68)	(4.47, 6.71)	(−12.79, −10.31)	(−7.65, −5.17)
	*p*-value	0.002	0.002	0.003	0.008	0.008

OHT: ocular hypertension; dLGN: dorsolateral geniculate nucleus; r: Pearson’s r; CI: 95% confidence interval.

**Table A10:** GFAP dLGN.

GFAP dLGN					
		Vehicle vs. CitiQ10	Vehicle vs. OHT dLGN Right	Vehicle vs. Contralateral dLGN Left	CitiQ10 vs. Contralateral-CitiQ10 dLGN Left
**Total**	Effect size |r|	0.66	0.81	0.77	
	CI (95%)	(2.261, 11.34)	(10.11, 33.04)	(8.138, 22.21)	
	*p*-value	0.007	0.0016	0.0031	
**Peripheral**	Effect size |r|	0.86	0.78	0.86	0.72
	CI (95%)	(1.053, 11.27)	(2.712, 31.09)	(8.245, 5.35)	(1.829, 26.67)
	*p*-value	0.007	0.0295	0.0031	0.0295
**Central**	Effect size |r|	0.86	0.85	0.83	
	CI (95%)	(1.285, 12.23)	(8.981, 33.67)	(7.240, 21.85)	
	*p*-value	0.0148	0.0016	0.0031	

OHT: ocular hypertension; dLGN: dorsolateral geniculate nucleus; r: Pearson’s r; CI: 95% confidence interval.

**Table A11:** Iba-1 SC.

Iba-1 SC							
		Vehicle vs. OHT SC Right	Vehicle vs. Contralateral SC Left	Citiq10 Vs. OHT-Citiq10 SC Right	CitiQ10 vs. Contralateral-CitiQ10 SC Left	OHT SC Right vs. OHT-CitiQ10 SC Right	Contralateral SC Left vs. Contralateral-CitiQ10 SC Left
**Total**	Effect size |r|	0.83		0.83			
	CI (95%)	(3.76, 6.24)		(6.76, 9.24)			
	*p*-value	0.008		0.008			
**Active morphotypes**	Effect size |r|	0.83	0.83	0.83	0.76	0.83	0.79
	CI (95%)	(15.91, 18.39)	(2.84, 5.32)	(10.00, 12.48)	(0.42, 2.90)	(−6.95,−4.47)	(−3.46, −0.982)
	*p*-value	0.008	0.008	0.008	0.016	0.008	0.16

OHT: ocular hypertension; SC: superior colliculus; r: Pearson’s r; CI: 95% confidence interval.

**Table A12:** GFAP SC.

GFAP SC						
	Vehicle vs. CitiQ10	Vehicle vs. OHT SC Right	Vehicle vs. Contralateral SC Left	CitiQ10 vs. OHT-CitiQ10 SC Right	CitiQ10 vs. Contralateral-CitiQ10 SC Left	OHT SC Right vs. OHT-CitiQ10 SC Right
Effect size |r|	0.86	0.86	0.86	0.86	0.86	0.86
CI (95%)	(2.268, 8.687)	(25.22, 31.70)	(7.253, 15.11)	(6.838, 16.01)	(2.291, 5.732)	(−16.99, −5.265)
*p*-value	0.0007	0.001	0.001	0.0025	0.0303	0.0079

OHT: ocular hypertension; SC: superior colliculus; r: Pearson’s r; CI: 95% confidence interval.

**Table A13:** Iba-1 V1.

Iba-1 V1								
		Vehicle vs. CitiQ10	Vehicle vs. OHT V1 Right	Vehicle vs. Contralateral V1 Left	CitiQ10 vs. OHT-CitiQ10 V1 Right	CitiQ10 vs. Contralateral-CitiQ10 V1 Left	OHT V1 Right vs. OHT-CitiQ10 V1 Right	Contralateral V1 Left vs. Contralateral-CitiQ10 V1 Left
**Active morphotypes**	Effect size |r|	0.83	0.83	0.83	0.83	0.83	0.83	0.83
	CI (95%)	(2.33, 4.81)	(5.35, 7.83)	(3.53, 6.01)	(−3.29, −0.81)	(−5.58, −3.10)	(−6.45, −3.97)	(−7.05, −4.57)
	*p*-value	0.008	0.008	0.008	0.008	0.008	0.008	0.008

OHT: ocular hypertension; V1: visual cortex; r: Pearson’s r; CI: 95% confidence interval.

**Table A14:** GFAP V1.

GFAP V1				
	Vehicle vs. CitiQ10	Vehicle vs. OHT V1 Right	Vehicle vs. Contralateral V1 Left	OHT V1 Right vs. OHT-CitiQ10 V1 Right
Effect size |r|	0.86	0.85	0.85	0.83
CI (95%)	(0.2333, 6.494)	(6.274, 15.54)	(1.799, 11.04)	(−19.06, −5.650)
*p*-value	0.0221	0.0043	0.0043	0.0079

OHT: ocular hypertension; V1: visual cortex; r: Pearson’s r; CI: 95% confidence interval.
